# P-stereocontrolled synthesis of oligo(nucleoside N3′→O5′ phosphoramidothioate)s – opportunities and limitations†‡

**DOI:** 10.1039/d0ra04987e

**Published:** 2020-09-23

**Authors:** Ewa Radzikowska, Renata Kaczmarek, Dariusz Korczyński, Agnieszka Krakowiak, Barbara Mikołajczyk, Janina Baraniak, Piotr Guga, Kraig A. Wheeler, Tomasz Pawlak, Barbara Nawrot

**Affiliations:** Centre of Molecular and Macromolecular Studies, Polish Academy of Sciences Sienkiewicza 112 90-363 Łódź Poland eradziko@cbmm.lodz.pl; Whitworth University, Department of Chemistry 300 W. Hawthorne Rd. Spokane WA 99251 USA

## Abstract

3′-*N*-(2-Thio-1,3,2-oxathiaphospholane) derivatives of 5′-*O*-DMT-3′-amino-2′,3′-dideoxy-ribonucleosides (_N_OTP-N), that bear a 4,4-unsubstituted, 4,4-dimethyl, or 4,4-pentamethylene substituted oxathiaphospholane ring, were synthesized. Within these three series, _N_OTP-N differed by canonical nucleobases (*i.e.*, Ade^Bz^, Cyt^Bz^, Gua^iBu^, or Thy). The monomers were chromatographically separated into P-diastereomers, which were further used to prepare N_NPS_N′ dinucleotides (3), as well as short P-stereodefined oligo(deoxyribonucleoside N3′→O5′ phosphoramidothioate)s (NPS-) and chimeric NPS/PO- and NPS/PS-oligomers. The condensation reaction for _N_OTP-N monomers was found to be 5–6 times slower than the analogous OTP derivatives. When the 5′-end nucleoside of a growing oligomer adopts a C3′-*endo* conformation, a conformational ‘clash’ with the incoming _N_OTP-N monomer takes place, which is a main factor decreasing the repetitive yield of chain elongation. Although both isomers of N_NPS_N′ were digested by the HINT1 phosphoramidase enzyme, the isomers hydrolyzed at a faster rate were tentatively assigned the *R*_P_ absolute configuration. This assignment is supported by X-ray analysis of the protected dinucleotide ^DMT^dG^iBu^_NPSMe_T_OAc_, which is P-stereoequivalent to the hydrolyzed faster P-diastereomer of dG_NPS_T.

## Abstract 2

Separated P-diastereomers of 3′-*N*-(2-thio-1,3,2-oxathiaphospholane) derivatives of 5′-*O*-DMT-3′-amino-2′,3′-dideoxy-ribonucleosides were used to prepare P-stereodefined N_NPS_N′ dinucleotides and short NPS-, NPS/PO- and NPS/PS-oligomers.

## Graphical abstract

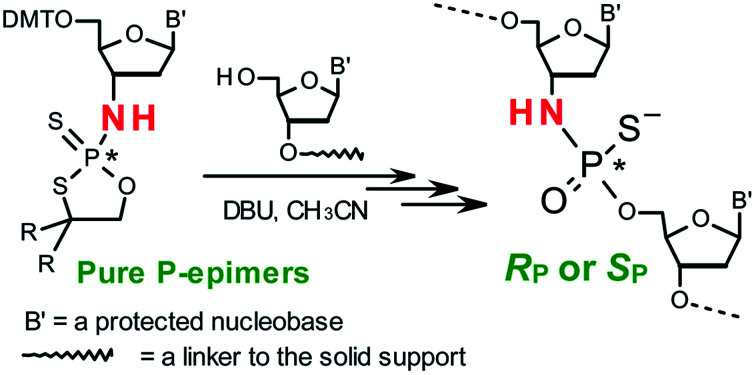

## Introduction

**Chart 1:**
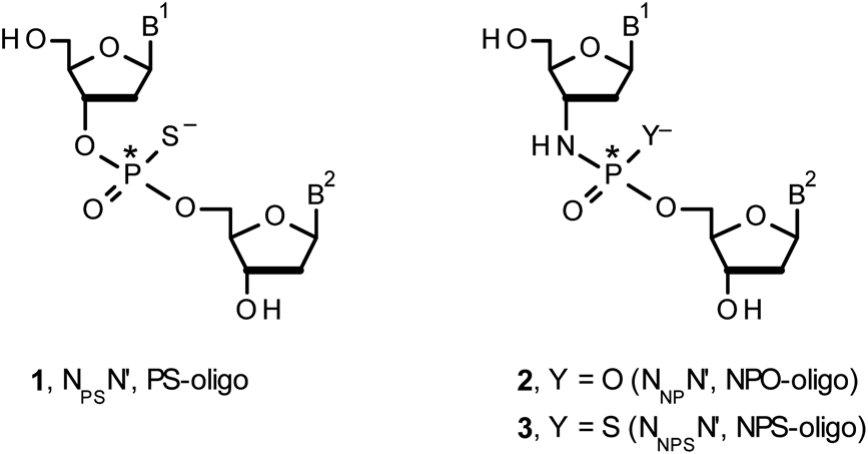
B^1^,B^2^ = Ade, Cyt, Gua or Thy. The asterisks indicate the P-stereogenic centers.

**Scheme 1:**
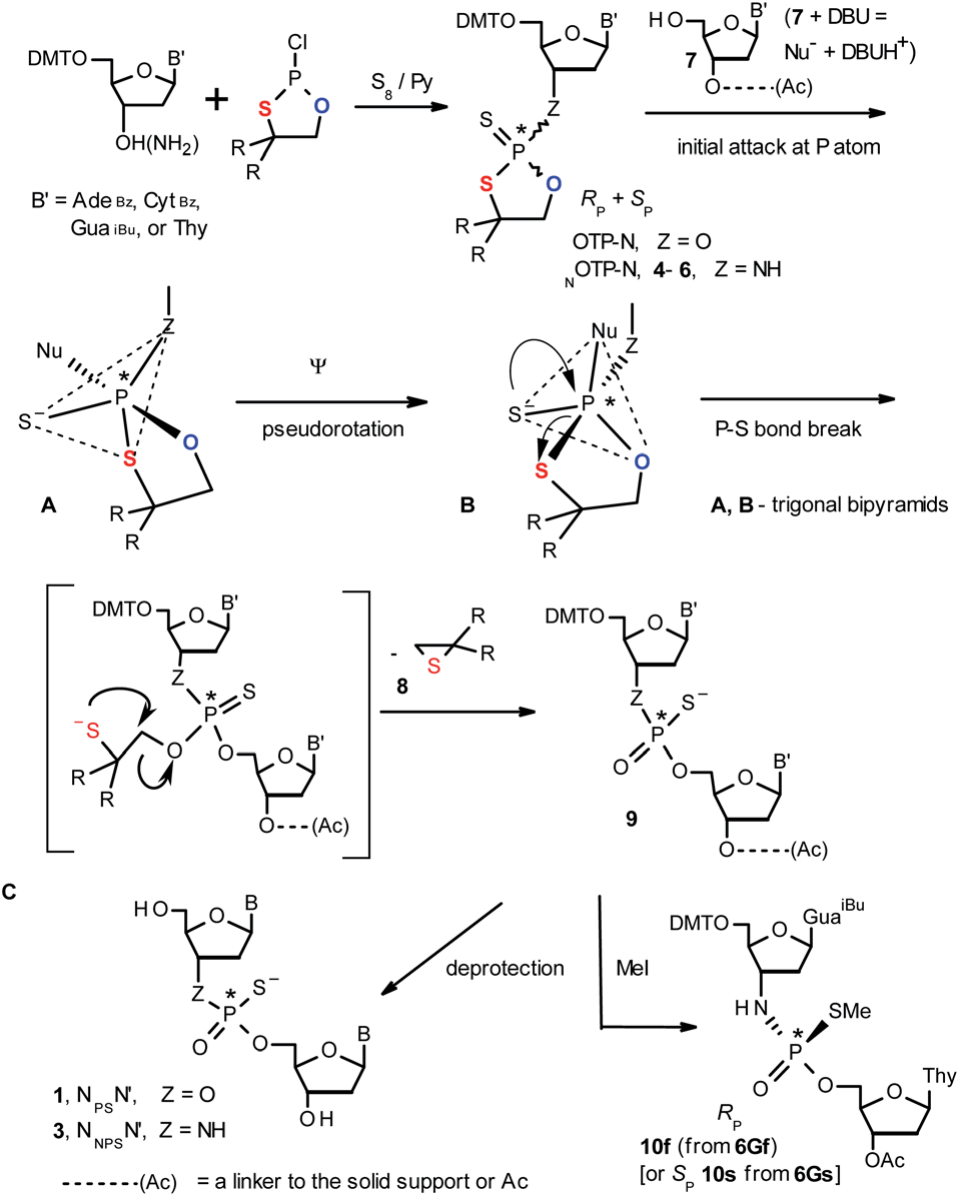
A mechanism of the DBU-promoted condensation step in Otp synthesis of N_PS_N′ or N_NPS_N′ with the use of a mixture of P-epimers of OTP-N (Z = O) or _N_OTP-N monomers (Z = NH), respectively. 4, R = H; 5, R = Me; 6, R,R = −(CH_2_)_5_–. B′ = Ade^Bz^, Cyt^Bz^, Gua^iBu^, or Thy. The codes 6Gf and 6Gs indicate fast- and slow-eluting P-diastereomers of *Pm*-_N_OTP-dG (*vide infra*).

Therapeutic properties of synthetic DNA oligonucleotides (PO-oligos) targeted against complementary DNA or mRNA molecules have been tested for more than 40 years.^1^ In principle, the probes should be characterized by high target affinity, efficient cellular uptake, and enhanced resistance towards nucleases. To make PO-oligos more stable towards nucleases, phosphorothioate analogs (PS-oligos, a dinucleotide N_PS_N′ (1) is shown in Chart 1) were introduced many years ago^2^ and remain of interest to the scientific community today.^2*e*^ A single PO→PS substitution performed with a non-bridging oxygen atom produces a new stereogenic center, thus PS-oligos synthesized by the standard, marginally stereoselective, phosphoramidite or H-phosphonate methods are mixtures of hundreds or thousands of P-diastereomers.^3^ To date, P-stereodefined PS-oligos have been mainly prepared by (developed in this laboratory) the oxathiaphospholane approach^4^ (Otp, see Scheme 1) based on the use of P-diastereomerically pure 5′-*O*-DMT-nucleoside-3′-*O*-(2-thio-1,3,2-oxathiaphospholane)s or their 4,4-disubstituted analogs (OTP-N; R = H or R = Me or R,R = −(CH_2_)_5_–, Scheme 1). Other methods are available as well.^5^ With the exception of homopurine *R*_P_-PS-oligos, which with the RNA complement(s) form thermally stable parallel duplexes and triplexes with overall A conformation,^6^ both *R*_P_- and *S*_P_-PS-oligos possess unfavorable hybridization properties as compared to those observed for the unmodified oligonucleotides.^7^ However, their physico-chemical^7–9^ and biological^10–12^ properties often depend on the absolute configuration of the P-atoms.

Another class of DNA analogs of considerable nucleolytic stability consists of P-achiral oligo(deoxyribonucleoside phosphoramidate)s^13^ (NPO-oligos, a dinucleotide N_NP_N′ (2) is shown in Chart 1), in which the 3′-oxygen atom is replaced by a nitrogen atom.^14^ CD measurements^15^ as well as high-resolution X-ray crystallographic data^16^ of duplexes formed by NPO-oligos and DNA or RNA strands revealed A-like conformations, which contribute to their high thermal stability.^15^ NPO-oligos were found to be allosteric inhibitors of telomerase, which is a ribonucleoprotein responsible for maintaining telomeres in nearly all eukaryotic cells.^17^

Oligo(deoxyribonucleoside phosphoramidothioate)s (NPS-oligos, a dinucleotide N_NPS_N′ (3) is shown in Chart 1 and in Scheme 1) were synthesized to combine the useful properties of PS- and NPO-oligos.^18^ NPS-oligos are P-chiral and the syntheses based on the widely used, yet of low stereoselectivity, phosphoramidite or H-phosphonate methodology generate stereorandom mixtures of P-epimers. Important biochemical findings about NPS-oligos§§Noteworthy, an NPS-oligomer bearing a lipid tag (GRN163L^50^) was found to be a potent inhibitor of human telomerase,^51^ which is an enzyme highly active in ∼85% of known human tumor cells, whereas in normal cells its activity is marginal. prompted us to check if P-stereodefined NPS-oligos may be obtained in a variant form of the Otp methodology using the monomers 4–6 (_N_OTP-N, Z = NH: 4, R = H; 5, R = Me; 6, R,R = −(CH_2_)_5_–; B′ = Ade^Bz^, Cyt^Bz^, Gua^iBu^, or Thy, Scheme 1). Importantly, it was documented,^19^ that if Z = O the Otp condensation does not follow a stereoinvertive S_N_2P mechanism, but this is a stereoretentive process, where the initial attack of a nucleophile 7 takes place from the side opposite to the most electronegative atom attached to the phosphorus center (the oxygen atom in OTP-N, marked in blue; Scheme 1) to form a trigonal bipyramid A. Then, the permutational isomerization (a pseudorotation process) furnishes the bipyramid B, where the leaving thioalkyl group occupies the axial position, necessary for the cleavage of the P–S bond. The condensation process concludes with the elimination of episulfide 8 from the triester intermediate C. It was assumed, that _N_OTP-N would react with the 5′-OH group of 7 in an analogous manner to yield phosphoramidothioate diester 9 (Z = NH), with a final deprotection step producing NPS-oligos 3.

**Chart 2:**
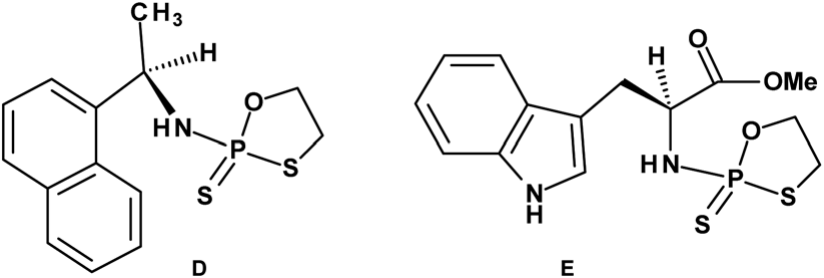
(D) (*R*_c_)-2-(1-(α-Naphthyl)ethyl)amino)-2-thio-1,3,2-oxathiaphospholane, (E) a 2-thio-1,3,2-oxathiaphospholane derivative of l-tryptophan methyl ester; _N_OTP-Trp.

Early studies in this field showed that both P-epimers of (*R*_c_)-2-(1-(α-naphthyl)ethyl)amino)-2-thio-1,3,2-oxathiaphospholane (Chart 2, structure D) in the presence of DBU reacted with primary alcohols to give products with stereochemical retention – *i.e.*, the incoming alkoxyl group was found in the product at the position originally occupied by the endocyclic sulfur atom (marked in red in Scheme 1).^20^

Several years ago such high stereoselectivity was confirmed in the reaction of the _N_OTP derivative of l-tryptophan methyl ester (_N_OTP-Trp, Chart 2, structure E) with *O*2′,*O*3′,*N*6-tribenzoyl-adenosine^21^ leading to the tribenzoylated derivative of AMPS-Trp-OMe. Similar stereoselective results were achieved from the reactions of P-diastereomers of the _N_OTP derivative of 3′-amino-3′-deoxy-thymidine (5, B′ = Thy) with 3′-*O*-acetyl-thymidine, which (after deprotection) furnished the T_NPS_T dimers.^22^

Once obtained, homopurine *R*_P_-NPS-oligos with the anticipated intrinsic C3′-*endo* conformation (*vide supra*) could be used to effectively stabilize the previously described parallel duplexes and triplexes.^6^

Here we show that using P-diastereomerically pure _N_OTP-N monomers results in P-stereodefined chimeric PO/NPS- or PS/NPS-oligos with the NPS-nucleotides introduced in “alternate” positions. Additional experiments revealed several structural factors responsible for the observed low repetitive yields in the solid phase synthesis of uniformly modified NPS-oligos.

## Results and discussion

### Preparation of P-diastereomerically pure _N_OTP-N monomers 4–6

Our earlier experiments with OTP-N monomers (Z = O, Scheme 1) showed that in terms of chromatographic separability of P-diastereomers, those 4,4-unsubstituted (R = H) were most troublesome, followed by the more convenient compounds bearing R = Me and the most useful 4,4-pentamethylene substituted congeners.^4^ The same was observed for their LNA (Locked Nucleic Acids) analogs.¶¶Unpublished results. The rank was determined during work on ref. 25.

**Scheme 2:**
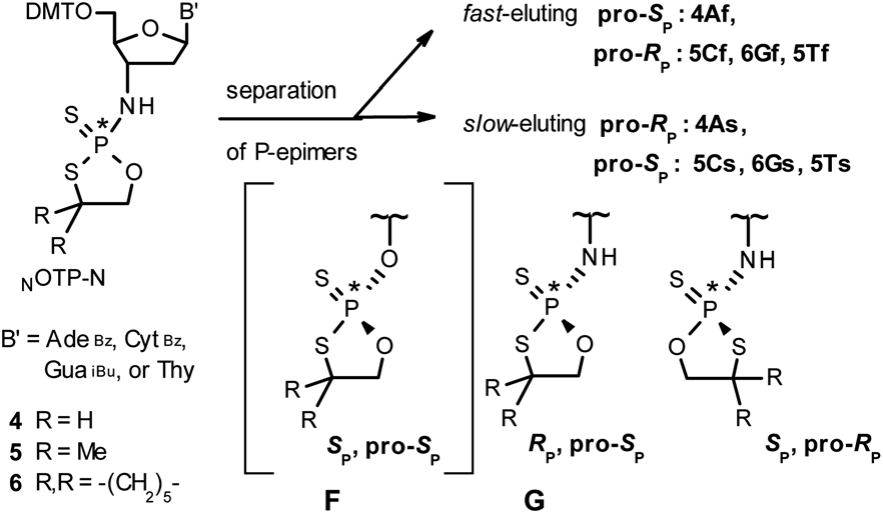
Effects of silica gel separation of P-diastereomers of 2-thio-1,3,2-oxathiaphospholane monomers 4–6. The descriptors pro-*R*_P_ and pro-*S*_P_ indicate the absolute configuration of P-atom in the subsequently formed internucleotide phosphoramidothioate linkages. The structure F is given only for comparison with G.

In the present work three sets of _N_OTP-N monomers (4,4-unsubstituted, *Un*-, 4; 4,4-dimethyl substituted, *Dm*-, 5; and 4,4-pentamethylene substituted, *Pm*-_N_OTP-N, 6; Scheme 2) were obtained from the 5′-*O*-DMT derivatives of 3′-amino-2′,3′-dideoxy-ribonucleosides. However, chromatographic separation of P-diastereomers was achieved only for 4A, 5T, 5C, and 6G (the suffixes A, G, C or T indicate the Ade, Gua, Cyt or Thy nucleobases, respectively). The chromatographic details related to the separation of the fast- and slow-eluting P-diastereomers are given in Table 1, and the relative mobility is reflected in their codes by an f or s suffix, respectively (*e.g.*4Af or 4As, Scheme 2). The relevant HR MS, ^31^P NMR, ^1^H NMR, and ^13^C NMR spectra are shown in Data Sets S1–S4 and Fig. S1–S4 (ESI).‡ Attempts to separate other _N_OTP-Ns, *e.g.*5A or 6A, were unsuccessful with the data for unresolved 4–6 given in Table S1 (ESI).‡ [Note: The pro-*R*_P_ and pro-*S*_P_ descriptors, shown in Scheme 2, indicate the absolute configuration of P-atom in the internucleotide phosphoramidothioate moiety formed upon condensation of a given P-diastereomer of _N_OTP-N with the 5′-OH group of a nucleoside/nucleotide. The assignment was based on the differences in rates of hydrolysis of P-diastereomeric N_NPS_N′ dinucleotides 3 (Scheme 1) with HINT1 phosphoramidase (*vide infra*)]. It should be noted, that stereochemically equivalent pro-*S*_P_ OTP-N and _N_OTP-N monomers (Scheme 2, F and G, respectively) have opposite absolute configurations of the phosphorus stereogenic centers. Obviously, the same relationship is valid for the pro-*R*_P_ pairs.

**Table 1:** Characteristics of separated P-diastereomers of _N_OTP-N 4–6

B^′^, R,R	Yield^a^ (%)	MW calc. (Da)	TOF MS ES (*m*/*z*)^b^	Code	*R*_f_ c	^31^P NMR^d^ (*δ*, ppm)
Ade^Bz^, H,H	83	794	795.2191; 817.2008 [M + Na^+^] 100%	4Af^i^	0.50	95.92
	24/12^e^			4As^i^	0.48^j^	95.25
Cyt^Bz^, Me,Me	79	798	799.2385	5Cf	0.70	97.74
	19/26^f^			5Cs	0.65^k^	97.25
Gua^iBu^, –(CH_2_)_5_–	84	844	845.2920	6Gf	0.58	96.92
	52/26^g^			6Gs	0.47^k^	96.94
Thy, Me,Me	88	709	732.1951, [M + Na^+^]	5Tf	0.65	96.69
	22/22^h^			5Ts	0.58^l^	96.58

a Total yield of the isolated mixture of isomers; yields of the separated fast- and slow-eluting isomers, respectively; ‘flash’ chromatography on silica gel 200–300 mesh was performed unless otherwise stated.

b [M + H^+^] ions, measured for the unresolved mixture of P-epimers.

c HP TLC plates.

d In CDCl_3_.

e Silica gel 60H, 0→1% MeOH/CHCl_3_, v/v.

f 0→2% MeOH/CHCl_3_, v/v.

g CHCl_3_ : MeOH 50 : 1, v/v.

h AcOEt : hexane 1 : 1, v/v.

i Only a minute amount of 4Af was isolated and in further experiments a 1 : 2 mixture of 4Af and 4As was used.

j CHCl_3_ : MeOH 20 : 1, v/v, double development.

k CHCl_3_ : MeOH 20 : 1, v/v.

l AcOEt : hexane 1 : 1, v/v.

### Tentative assignment of the absolute configuration at P-atoms in separated diastereomers of _N_OTP-Ns 4–6

During the course of earlier works on stereocontrolled synthesis of PS-oligos, the experiments of enzymatic hydrolysis of dinucleoside phosphorothioates N_PS_N′ (1) with *R*_P_-specific snake venom phosphodiesterase (svPDE) and *S*_P_-specific Nuclease P1 (ref. 23) provided sufficient data to establish an empirical rule that the fast-eluting *Un*-OTP-N (Z = O, B’ = Ade^Bz^, Cyt^Bz^, Gua^iBu,DPC^, or Thy) are precursors of *S*_P_-N_PS_N′, contrary to fast-eluting *Dm*- and *Pm*-OTP-N congeners, which yield *R*_P_-N_PS_N′.^4*a*,19^ This relationship was later confirmed for the *Pm*-OTP derivatives of LNA nucleosides.^24,25^ Unfortunately, although NPO-oligos were hydrolyzed by svPDE to a measurable extent (after 4.5 h incubation ∼50% of NPO-T_10_ remained intact;^13*a*^ after 24 h incubation phosphoramidate T_NP_T was hydrolyzed in 22% yield, data not shown), phosphoramidothioates 3 when treated with svPDE or Nuclease P1 remained intact.

**Chart 3:**
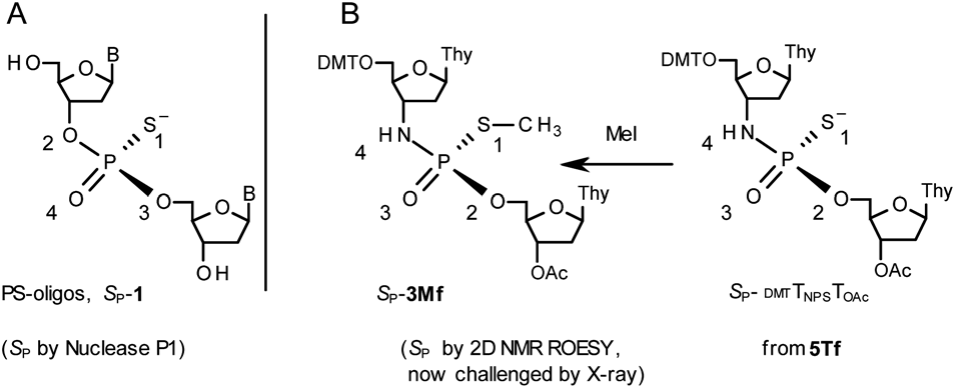
Orientation of the sulfur, phosphoryl oxygen and thymidine O5′ atoms around the phosphorus atoms: Panel A, in phosphorothioate *S*_P_-1, ref. 23; Panel B, 2D NMR ROESY based assignment (here challenged by X-ray analysis of 10f, Scheme 1) in the *S*-methylated *S*_P_-3Mf, ref. 22 (the phosphoramidothioate diester precursor was obtained from fast-eluting 5Tf). The Arabic numerals 1–4 indicate the priority of substituents around the phosphorus atoms according to the Cahn–Ingold–Prelog rules. Note: to establish the priority of substituents, the formal double bond between the phosphoryl oxygen atom and the phosphorus atom (P=O) should be considered a single one.

A few years ago, chromatographically separated 5Tf and 5Ts (R = Me, B′ = Thy) and 3′-*O*-acetylated thymidine were used in the synthesis of P-diastereomeric ^DMT^T_NPS_T_OAc_ (5′-*O*-DMT and 3′-*O*-Ac protected precursors of 3, Chart 3).^22^ Both anionic ^DMT^T_NPS_T_OAc_ diesters were then stereoretentively *S*-methylated to yield the corresponding P-diastereomeric triesters ^DMT^T_NPSMe_T_OAc_ (3Mf and 3Ms, Chart 3). At that point, 2D NMR ROESY experiments suggested that, contrary to the aforementioned empirical rule, the relative orientation of the sulfur, phosphoryl oxygen and thymidine O5′ atoms around the phosphorus atom in the *S*-methylated triester 3Mf (Chart 3, obtained from the fast-eluting 5Tf) is equivalent to that in phosphorothioate *S*_P_-1, which is hydrolyzed by Nuclease P1. Consequently, 3Ms obtained from the slow-eluting 5Ts is P-stereoequivalent to *R*_P_-1, which is hydrolyzed by svPDE. Although the O3′→N3′ replacement does change the priority of substituents (O3′ – priority 2, N3′ – priority 4), by virtue of the formalism of the Cahn-Ingold-Prelog rules, not only the relative orientations but also the absolute configurations of P atoms in *S*_P_-1, and 3Mf are the same.

**Fig. 1:**
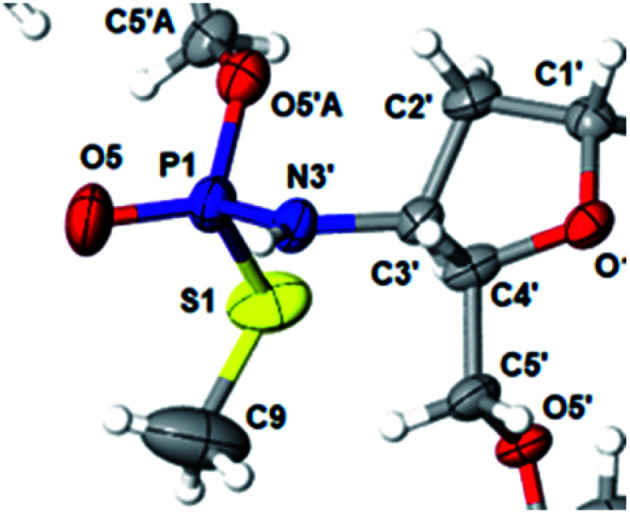
Crystal structure of ^DMT^dG^iBu^_NPSMe_T_OAc_10f showing partial structure and spatial orientation of substituents around the phosphorus stereogenic center (marked P1). Compound 10 was obtained from the monomer 6Gf (fast-eluting 5′-*O*-DMT-3′-amino-2′,3′-dideoxy-*N*6-iBu-guanosine-3′-*N*-(2-thio-4,4-pentamethylene-1,3,2-oxathiaphospholane). The oxygen atom marked O5′A belongs to the thymidine residue.

This violation of the empirical rule “fast-eluting *Dm*- and *Pm*-OTP-N → *R*_P_-N_PS_N” could not be explained by the C3′-*endo* conformation of the sugar moiety in 3′-amino-2′,3′-dideoxy-nucleosides, because the C3′-*endo* locked LNA-derived oxathiaphospholane monomers adhere to that rule.^24^ In the present work, this unexpected NMR-based assignment was challenged by a successful crystallographic experiment, which showed the rule-obeying *R*_P_ absolute configuration (Fig. 1) for a non-ionic ^DMT^dG^iBu^_NPSMe_T_OAc_ amidodiester (10f, Scheme 1, ESI‡) obtained from the fast-eluting 6Gf (a *Pm*-derivative) after S-methylation of the negatively charged amidoester intermediate ^DMT^dG^iBu^_NPS_T_OAc_ (9). Importantly, compound 10f crystallized spontaneously, without the typically applied slow evaporation. The complete crystal structure is shown in Fig. S7 (ESI)‡ with crystallographic details summarized in Table S2 (ESI).‡

**Scheme 3:**
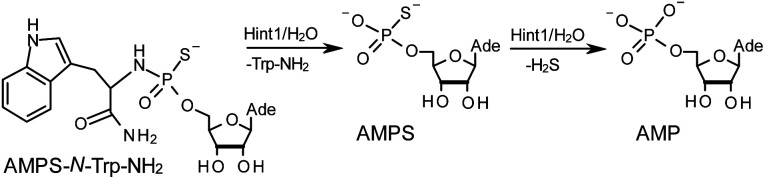
The HINT1 catalyzed hydrolysis of adenosine-5′-*O*-((l-tryptophan amide-*N*α-yl)-phosphoramidothioate) (AMPS-Trp-NH_2_), followed by desulfuration of the AMPS intermediate leading to the formation of adenosine-5′-*O*-phosphate.

The opposite absolute configurations of P-atoms determined by the dinucleotide derivatives 3Mf and 10f, obtained from the fast-eluting *Dm*-5Tf (the NMR data) and from the fast-eluting *Pm*-6Gf (the X-ray data), respectively, seemed to be a contradiction. Although it is possible that the *Dm*-_N_OTP monomers, unlike *Dm*-OTP monomers, behave differently from the *Pm*-analogs, the contradiction could not be left unaddressed. Because attempts at crystallization of other N_NPS_N′ dimers (protected or unprotected) or their _N_OTP-N precursors were unsuccessful, we turned our attention to HINT1 phosphoramidase. This enzyme belongs to a family of hydrolases and transferases characterized by the presence of the histidine triad H–X–H–X–H–X–X–X (HIT) motif at their catalytic center, where H is a histidine residue and X is a hydrophobic amino acid.^26^ The HINT1 catalyzed *in vitro* hydrolysis of the P–N bond releases (d)NMP or (d)NMPS from their phosphoramidate or phosphoramidothioate conjugates with amino acids, *e.g.*, from a conjugate of l-tryptophan amide and AMPS (AMPS-Trp-NH_2_||||AMPS-Trp-NH_2_ was concomitantly obtained from AMPS-Trp-OMe during ammonolysis of the protecting benzoyl groups in the adenosine moiety., Scheme 3).^21,27^

It is known that the hydrolysis of the P–N bond in the *R*_P_ isomer of AMPS-Trp-NH_2_ is approximately 4 times faster than the same reaction using the *S*_P_ counterpart.^21^ The released (d)NMPS is further desulfured to form (d)NMP and hydrogen sulfide, and this secondary nucleotide product must be taken into account during HPLC-based quantification of the products of hydrolysis. The rates of desulfuration of (d)NMPS decrease in the following order: AMPS, GMPS ≥ CMPS > UMPS > dAMPS, dGMPS ≫ dCMPS, TMPS.^28^ One can assume that this decreasing order of affinity would also be reflected in the rates of hydrolysis of the *R*_P_ and *S*_P_ series of (d)NMPS-conjugates. To determine the absolute configuration at P-atoms in the oligomers obtained from the separated fast- and slow-eluting P-diastereomers of 4–6, model N_NPS_N′ dimers 11–17 (see Table 2) were prepared using the solid phase synthesis method. Due to the small difference of *R*_f_ values, only a minute amount of 4Af was isolated and in further experiments a 1 : 2 mixture of 4Af and 4As was used. [Notes: (a) To prevent the DBU-promoted intramolecular cleavage of the standard LCAA (long chain alkylamino) linker, a modified solid-phase support (-$) was used, in which the 3′-OH group of nucleoside was attached to the CPG (controlled pore glass) beads *via* a sarcosine linker [–COCH_2_CH_2_CON(CH_3_)CH_2_CO–LCAA–CPG].^29^ (b) To assess relative reactivity of _N_OTP-N and OTP-N monomers, ^HO^dG-$ was elongated with an equimolar mixture of _N_OTP-T and OTP-T, and the resultant mixture consisted of T_NPS_dG and T_PS_dG at *ca.* 1 : 5 ratio.]

**Table 2:** Coupling yields [%] in solid phase synthesis of the NPS-dimers 11–17 calculated from the DMT^+^ cation measurement. The descriptions fast and slow refer to the relative chromatographic mobility of _N_OTP-N monomers

_N_OTP-N	d(N_NPS_N′)	_N_OTP-N substrate	
		Fast	Slow
4A	11 d(A_NPS_A)		73^a^
5C	12 d(C_NPS_A)	98	95
6G	13 d(G_NPS_G)	75	46
5T	14 T_NPS_dA	93	89
5T	15 T_NPS_dG	98	95
5T	16 T_NPS_T	99	96
6G	17 dG_NPS_T	84	69

a For a 1 : 2 mixture of 4Af and 4As, see note *i* to Table 1.

**Fig. 2:**
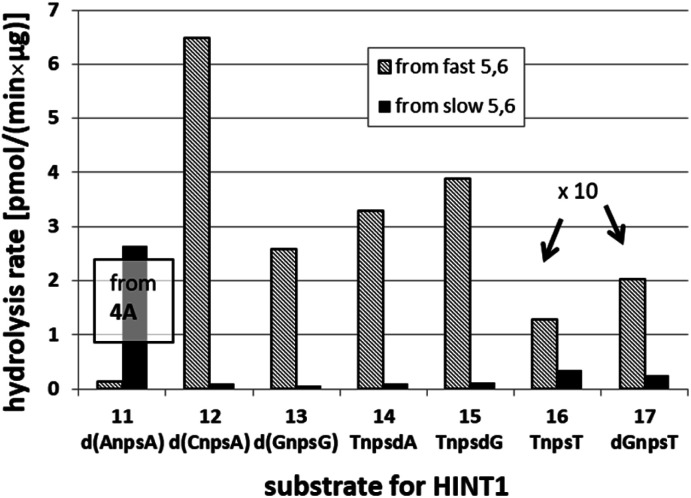
Rates of hydrolysis of NPS-dinucleotides 11–17 (data from Table 2). The descriptions fast and slow refer to the relative chromatographic mobility of _N_OTP-N precursors. For better visualization the heights of bars depicting the rates of hydrolysis of 16 and 17 have been scaled up by a factor of 10.

We recognized that the d(N_NPS_N′) dimers, as well as T_NPO_T, were hydrolyzed by recombinant human HINT1 phosphoramidase. In an initial rate assay, the substrates were treated at 37 °C until 10% degradation was detected by RP HPLC. The observed hydrolysis rates are given in Fig. 2 and Table 3. [Note: because the phosphoramidate conjugates of TMPS are the least effective substrates for HINT1 (*vide supra*) the rates of hydrolysis of 16 and 17 were very low; therefore, for better visualization the corresponding four bars in Fig. 2 were increased by a factor of 10.] Assuming that the hydrolysis of N_NPS_N′ mechanistically follows that observed for AMPS-Trp-NH_2_, the *R*_P_-N_NPS_N′ dimers should be hydrolyzed faster than the *S*_P_ counterparts. Based on this assumption, the HPLC analysis allowed assignment of the *R*_P_ absolute configuration to d(A_NPS_A) obtained from slow-eluting 4As, and to d(C_NPS_A), T_NPS_dA, T_NPS_dG, T_NPS_T, dG_NPS_T, and d(G_NPS_G) obtained from fast-eluting 5Cf, 5Tf, and 6Gf. This conclusion is contradictory to the earlier assignment from NMR data, but is consistent with the result of X-ray analysis shown in Fig. 1. Since the NMR based assessment of absolute configuration is less reliable than the crystallographic analysis, we claim that the resolved _N_OTP-N monomers follow the earlier observations made for OTP-N, so the fast-eluting 4Af is a precursor of *S*_P_-N_NPS_N′, and fast-eluting *Dm*- and *Pm*-_N_OTP-N yield *R*_P_-N_NPS_N′.

**Table 3:** Hydrolysis rates [pmol (min × μg protein)^−1^] found in the HINT1 catalyzed cleavage of the N_NPS_N′ 11–17 and the reference T_NPO_T (the initial rate assay). The descriptions fast and slow refer to the relative chromatographic mobility of the starting _N_OTP-N monomers. Each value represents the mean ± SD from at least three measurements

_N_OTP-N	d(N_NPS_N′)	_N_OTP-N substrate	
		Fast	Slow
4A	11 d(A_NPS_A)^a^	0.135 ± 0.007	2.65 ± 0.30
5C	12 d(C_NPS_A)	6.48 ± 0.36	0.107 ± 0.025
6G	13 d(G_NPS_G)	2.585 ± 0.019	0.063 ± 0.007
5T	14 T_NPS_dA	3.296 ± 0.631	0.099 ± 0.010
5T	15 T_NPS_dG	3.883 ± 0.651	0.125 ± 0.023
5T	16 T_NPS_T	0.129 ± 0.160	0.034 ± 0.007
6G	17 dG_NPS_T	0.202 ± 0.044	0.026 ± 0.004
NA	T_NPO_T	7.171 ± 1.225	

a Because a 1 : 2 mixture of 4Af and 4As was used in synthesis of 11, the corresponding 1 : 2 mixture of P-diastereomers was formed and P-diastereomerically pure 11 were obtained by RP HPLC separation of the fully deprotected compounds.

### Factors affecting the yield of condensation using _N_OTP-N

The previous experiments performed in our laboratory showed that, although the yield of the first coupling step of 6T (a mixture of P-diastereomers) with the 5′-OH group of a deoxyribonucleoside attached to a solid support was acceptable (*ca.* 90%), the yields associated with the subsequent coupling steps decreased at least by half.^30^ No improvement was observed when longer reaction times (600 s) or double delivery of the monomer (the second delivery after washing and drying of the support) were applied.

#### Solvent-related factors

**Fig. 3:**
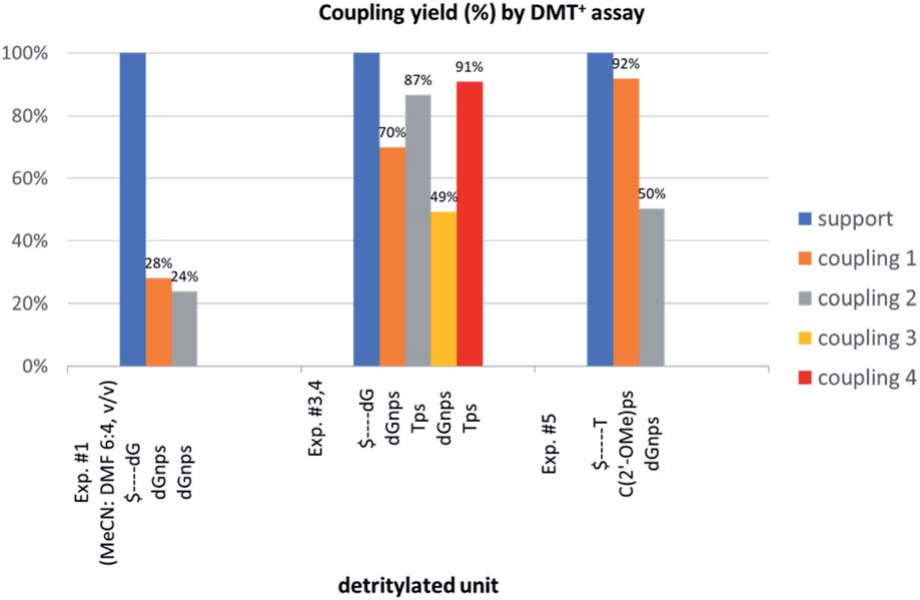
Coupling yields in the consecutive condensation steps, assessed by DMT^+^ assay at 504 nm. Absorptions of the DMT^+^ cation released from the nucleosides attached to the support (blue bars) are taken as 100%.

We decided to assess whether this failure was caused by poor solvation of the growing anionic ^HO^N_NPS_N′… oligomer in aprotic CH_3_CN, resulting in its poor “solubility” and restricted accessibility of the 5′-OH group (the reacting nucleophile) for the reagents. In an attempt to improve the solvation we used highly polar DMF, although this co-solvent did not help when the 2-oxo-4,4-pentamethylene-1,3,2-oxathiaphospholane analog of 6T was used in the synthesis of NPO-oligos.^30^ Unfortunately, when ^HO^dG-$ or ^HO^d(G_NPS_G)-$ were elongated with _N_OTP-dG (6G) in a CH_3_CN/DMF mixture (6 : 4, v/v) the condensation yield dropped even more (25–30%, assessed by the DMT^+^ assay, Fig. 3, Exp. #1), whereas the condensation with OTP-N (R = H) in the same solvent mixture was >90% effective (data not shown).

Because chemically NPS-oligos and PS-oligos differ by the presence of a 3′-NH amide group (a potent donor of hydrogen bonding) instead of 3′-O ester atom (a weak acceptor), one might assume that the former may be involved in aggregation, which potentially may be disrupted by the coordination of the 3′-NH with a strong non-nucleophilic amine. Because DBU is a sterically hindered amine and perhaps unable to provide this coordination, we tried to use a mixture of DBU and other less sterically demanding amines. The coupling reactions were performed at the 1 μmol scale (in all experiments thymidine was attached to the support) using 20-fold molar excess of each of four _N_OTP-N monomer 4, 50-fold excess of DBU and, starting from the second coupling, 50-fold excess of the additional amine. Unexpectedly, we noted a significantly increased repetitive yield of condensation only for 4T (67–88%, assessed by the DMT^+^ cation assay, Table 4). For unknown reasons, the best and the worst results were obtained using NEt_3_ and structurally similar NBu_3_, respectively. It should be noted that although in the past triethylamine was found to promote the condensation of much more reactive OTP-N monomers, the rate of coupling was low and several hours were necessary to complete the process.^19^

**Table 4:** Repetitive yield of coupling and overall yield of manual solid-phase synthesis (at 1 μmol scale) of the T_NPS_T_NPS_T_NPS_T oligomer using a monomer 4T and a mixture of DBU and given amine. The yields are derived from the DMT^+^ cation assay

Amine	p*K*_a_	Repetitive yield (%)	Overall yield (%)
NBu_3_	10.89	67	33
NEt_3_	10.76	88	68
DIPEA	10.50	76	44
DMAP	9.20	78	48
Collidine	7.48	74	36

#### Conformation-related factors

Data provided in Table 2 indicate that the first condensations with a solid support bound DNA nucleoside proceeded with required 90+% efficacy only for pyrimidine monomers 5T and 5C, while 6G offered the lowest coupling yield with, both T and dG bound to the support.**It must be pointed out that all the supports used were loaded with a given nucleoside to a similar extent (29–33 μmol g^−1^), and before the first condensation the support was extensively capped (Ac_2_O/DMAP/2,6-lutidine/tetrahydrofuran) to exclude unspecific coupling. To gain a more general understanding of this process, we extended this assay to all synthesized monomers, although the unresolved mixtures of P-diastereomers were used and coupling conditions were not optimized. In principle, these tendencies are seen for all investigated _N_OTP-N (Table 5), *i.e.*, the pyrimidine monomers are more reactive than purine ones.

**Table 5:** Average yield of the two consecutive coupling steps (%) in synthesis of N_NPS_N_NPS_N utilizing the unresolved _N_OTP-N monomers of different type

_N_OTP-N	Nucleobase			
	Ade^Bz^	Cyt^Bz^	Gua^iBu^	Thy
4	78	86	84	88
5	21	71	63	81
6	8	60	40	66

**Fig. 4:**
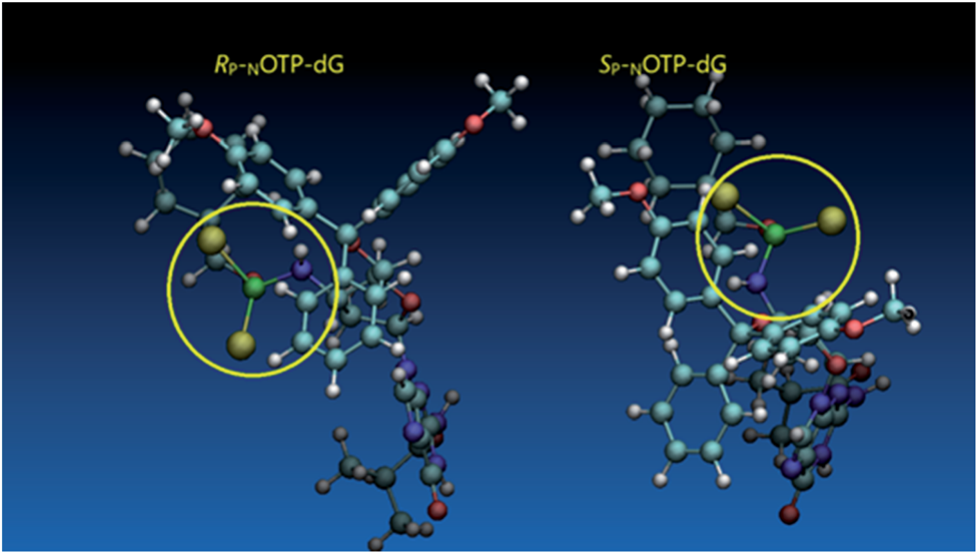
Visualization of the lowest energy conformers of 6G (*R*_P_*Pm*-_N_OTP-dG and *S*_P_*Pm*-_N_OTP-dG). The data were obtained using the Gaussian 16 package. Heteroatom labeling: P-green, O-red, S-yellow, N-navy blue.

Additionally, increasing the steric bulk of C4 substituents on the oxathiaphospholane ring results in a decrease in coupling efficiency. This phenomenon was most profound for 4A, 5A, and 6A, and using 6A the trimer d(A_NPS_A_NPS_A) was obtained in less than 10%. Fortunately, much more reactive 4A can be resolved onto P-epimers. On the contrary, 4G and 5G are much more reactive than 6G, but only the epimers of the latter monomer (6Gf and 6Gs) are separable. Interestingly, there are significant differences in the coupling yields for 6Gf and 6Gs (Table 2, 75% *vs.* 46%, 84% *vs.* 69%) and this observation prompted us to perform some calculations (using a Gaussian 16 software (ref. 31)) to assess their geometries. As expected, it was found that both compounds predominantly exist in a C3′-*endo* conformation. Then we focused our attention on the accessibility of the phosphorus centers for the initial attack of the nucleophile leading to the formation of the bipyramid A (Scheme 1). The views along the P–O bonds (the P and O atoms are green and red, respectively) are presented in Fig. 4. Comparing the areas inside the yellow circles one can notice that the steric hindrance is much lower in *S*_P N_OTP-dG, so the more reactive 6Gf can be tentatively assigned the *S*_P_ absolute configuration. Consequently, 6Gf should be a precursor for *R*_P_-N_NPS_N′ dinucleotides. This assignment is consistent with the crystallographic data collected for 10f and with the results of Hint1 promoted hydrolysis of 13 and 17, but cannot be considered decisive.

**Fig. 5:**
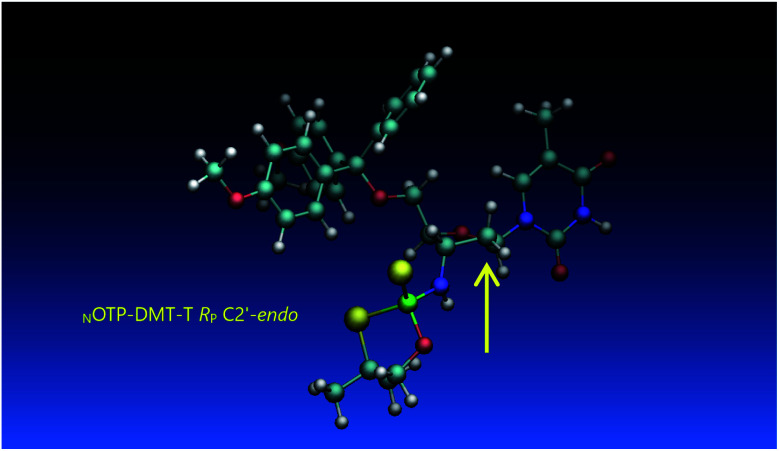
Visualization of the lowest energy conformer of *R*_P_-5T (the C2′-*endo* atom is marked with an arrow). The data were obtained using the Gaussian 16 package. Heteroatom labeling: P-green, O-red, S-yellow, N-navy blue.

Working earlier with LNA-derived OTP monomers (OTP-N^LNA^, which adopt the profound C3′-*endo* conformation), we observed (Exp. #2) that in the synthesis of per-(PS-LNA) oligonucleotides even double condensation of OTP-N^LNA^ to ^HO^N^LNA^_PS_…-$ was ineffective and a DMT^+^ cation absorption virtually decayed after the 5^th^ cycle (K. Jastrzębska, P. Guga, unpublished data); whereas, such a condensation with the 5′–OH–DNA-$ proceeded with >94% efficacy.^24^ As mentioned earlier, molecular modeling showed that poorly reacting 6Gf and 6Gs predominantly exist in a C3′-*endo* conformation. To verify whether the most reactive _N_OTP-T monomers 5Tf and 5Ts adopt that conformation (characteristic of 3′-amino-2′,3′-dideoxyribonucleosides), we performed NMR analysis. Unexpectedly, the recorded 2D ^1^H–^1^H COSY and ^1^H–^13^C EDITED-HSQC spectra showed ^3^*J*_H1′,H2′_ = 6.5 Hz, which according to literature data^32^ indicate a pseudorotation phase *P* = 90° or 195°. The former value is characteristic of a rare O4′-*endo* conformer, whereas the latter indicates a C2′-*endo* structure. To distinguish these options, a ^3^*J*_H3′,H4′_ value would be useful, but its measurement was significantly more complicated and neither analysis of the multiplicity of H3′ and H4′ signals nor attempts at simulation of the full spin system were successful. The molecular modeling experiments performed for *R*_P_-5T (5Ts, slightly less effective than 5Tf as shown in Table 2) showed that starting with C2′-*endo*, C3′-*endo*, or O4′-*endo* conformations the geometry optimization always ended at the C2′-*endo* conformation (Fig. 5), characteristic of the OTP-N monomers.

**Fig. 6:**
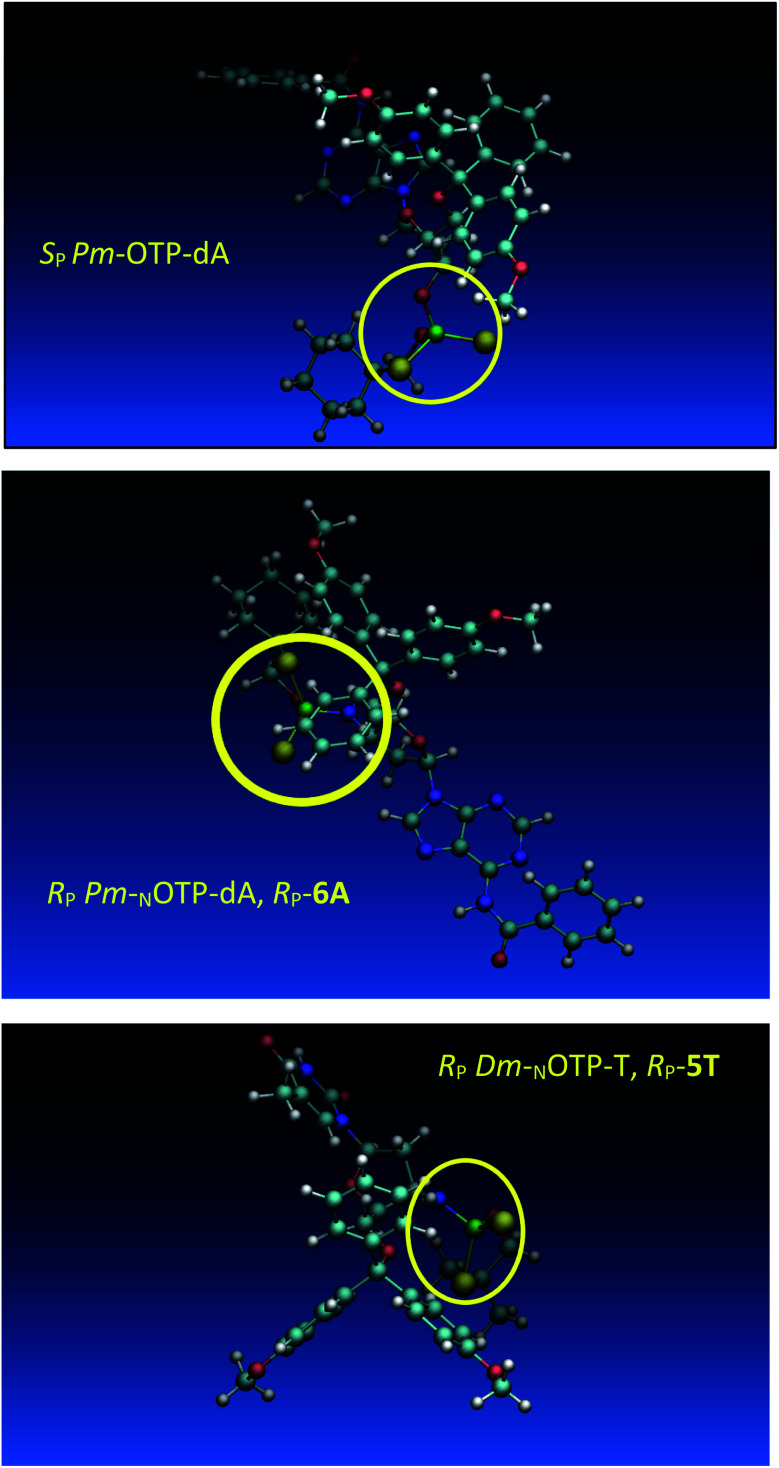
Predicted by molecular modeling the lowest energy conformations of: *S*_P_*Pm*-OTP-dA, an upper panel; *R*_P_-6A, a middle panel; *R*_P_-5T, a bottom panel. All three compounds are P-stereochemically equivalent (*vide supra*).

Analogous calculations were performed for (*S*_P_)-5′-*O*-DMT-*N*6-benzoyl-2′-deoxyadenosine-3′-*O*-(2-thio-4,4-pentamethylene-1,3,2-oxathiaphospholane) (*S*_P_*Pm*-OTP-dA, slightly less reactive than the *R*_P_ counterpart), and (*R*_P_)-5′-*O*-DMT-*N*6-benzoyl-3′-amino-2′,3′-dideoxy-adenosine-3′-*N*-(2-thio-4,4-pentamethylene-1,3,2-oxathiaphospholane) (*R*_P_*Pm*-_N_OTP-dA, *R*_P_-6A, the least effective monomer as shown in Table 5). [Note: looking along the P–O3′ bond in *S*_P_*Pm*-OTP-dA and along the P–N3′ bond in *R*_P_-6A, despite of the opposite absolute configurations of phosphorus atoms (*S*_P_*vs. R*_P_), all other substituents attached to the P-atoms are positioned with similar spatial orientations, so that both compounds are P-stereochemically equivalent.] Independent of the implemented solvent (acetonitrile or chloroform), the results were very similar and showed (see the yellow circles) that the approach of the nucleophile along the P–O bond in *S*_P_*Pm*-OTP-dA is virtually unrestricted (Fig. 6, an upper panel). Importantly, a similar marginally restricted approach is predicted for efficiently reacting 5Ts (Fig. 6, a bottom panel). On contrary, in a poorly reacting C3′-*endo* (*R*_P_)-3′-amino-2′,3′dideoxy-adenosine analog (a middle panel) such the approach is substantially blocked by the DMT propeller.

### Indications of ‘conformational clash’

In next three condensation experiments we used monomers of low coupling potency, *i.e.*, a 6Gf + 6Gs mixture. If ^HO^T_PS_d(G_NPS_G)-$ is condensed with an _N_OTP-G monomer (Exp. #3), the product is formed in 49% yield (Fig. 3), while only 40% yield was noted for elongation of ^HO^d(G_NPS_G)-$ (Table 5). If ^HO^d(G_NPS_G)-$ or ^HO^d(G_NPS_T_PS_G_NPS_G)-$ are elongated using an OTP-T monomer (C2′-*endo*) the efficacy is close to 90% (Exp. #4). If ^HO^C^(2’-OMe)^_PS_T-$ (the 2′-OMe unit exists in the C3′-*endo* conformation) reacts with an _N_OTP-dG monomer (Exp. #5), *ca.* 50% efficacy is observed.

Important information comes from a paper by Hodgson and co-workers, which describes ^1^H NMR conformational analysis of T_NP_^S^T dinucleotide, which is an anionic *S*-alkyl phosphoramidothiolate compound bearing a 5′-deoxy-5′-thio-thymidine residue (^S^T) at the 3′-end.^33^ They found that the 3′-amino-2′,3′-dideoxy-ribose ring adopts the C3′-*endo* conformation, while the ring of the ^S^T unit retains the C2′-*endo* conformation. Thus, the C3′-*endo* conformation is not transmitted downstream to the ^S^T DNA unit and the preserved neighboring conformations C3′-*endo*/C2′-*endo* lead to the lowest overall energy of the T_NP_^S^T system. This indicates that the C3′-*endo*/C2′-*endo* conformation of both nucleosides is energetically favored over the C3′-*endo*/C3′-*endo* conformation, which is observed in RNA/DNA hybrids. This observation may explain the greater reaction efficiency of _N_OTP-N with a growing oligomer bearing the 5′-end DNA unit (the first coupling step and the result of Exp. #3). At this point, one can mention ^DMT^dG^iBu^_NPSMe_T_OAc_ (10f), which also adopted the C3′-*endo*/C2′-*endo* conformation (Fig. S8, ESI‡), and spontaneous crystallization of which indicates remarkably low energy of this system.

Thus one can conclude that if the 5′-end segment of a growing oligomer (^HO^N_NPS_…-$, ^HO^N^LNA^_PS_…-$ in Exp. #2, or ^HO^C^(2’-OMe)^_PS_…-$ in Exp. #5) and an incoming monomer (_N_OTP-N or OTP-N^LNA^) adopt the C3′-*endo* conformation there is a more crowded space around the phosphorus atom. This conformational ‘clash’ may result in a decrease of reaction rate that promotes additional side-reactions and may heighten the damaging effect of the aforementioned intrinsic lower reactivity of the thiophosphoramide _N_OTP-N monomers compared to OTP-N. This decrease of coupling efficiency is much less severe if the incoming monomer exists in the C2′-*endo* conformation (Exp. #4 and relatively high reactivity of 5T).

### Attempts at solid phase synthesis of P-stereodefined NPS-oligo and chimeric NPS/PS and NPS/PO oligomers

The low repetitive yields encountered in the syntheses of N_NPS_N_NPS_N with a random configuration of phosphorus atoms (Table 5) were confirmed (by the DMT^+^ assay) by use of resolved P-epimers (see the column “Yield” in Table 6 for several examples). This circumstance rendered the syntheses of longer P-stereodefined NPS-oligo difficult. Also, to avoid extensive hydrolysis of phosphoramidothioate linkages upon treatment with 50% aqueous acetic acid^34^ at the detritylation step, performed after the otherwise beneficial DMT-ON RP HPLC purification, the last step of the solid support synthesis included the use of an anhydrous 3% solution of DCA in methylene chloride for detritylation. In the case of short oligomers 18–20 (up to heptamers), careful RP HPLC purification furnished the products of satisfactory purity, as assessed by PAGE and MALDI-TOF MS analyses (data not shown). The earlier mentioned lower efficiencies of condensation for the purine monomers, as well as those for the slow-eluting compounds, were confirmed in the syntheses summarized in Table 6. Only homo-thymidine oligomers 19 and 20 were isolated in appreciable amounts, while the samples of 18f and 18s allowed only for MALDI-TOF MS analysis.

**Table 6:** NPS-Oligos synthesized at 1 μmol scale using the _N_OTP-N and isolated by RP HPLC

Sequence	Code	_N_OTP-N substrate	Yield^a^ (%)	Amount^b^ (OD)
d(G_NPS_G_NPS_G)	18f	6Gf	17	0.5
	18s	6Gs	8	0.5
T_NPS_(T_NPS_)_4_T	19f	5Tf	37	4.2
	19s	5Ts	26	3.0
T_NPS_(T_NPS_)_5_T	20m	5T^c^	16	9.8
T_NPS_(T_NPS_)_8_T	21m	5T^c^	21	5.4
	21f	5Tf	17	7.5

a The yield calculated from the absorbance measured (at 504 nm) for the last dimethoxytrityl cation released compared to the initial value.

b After double HPLC purification on a C18 reverse phase column.

c An unresolved mixture of P-epimers.

**Fig. 7:**
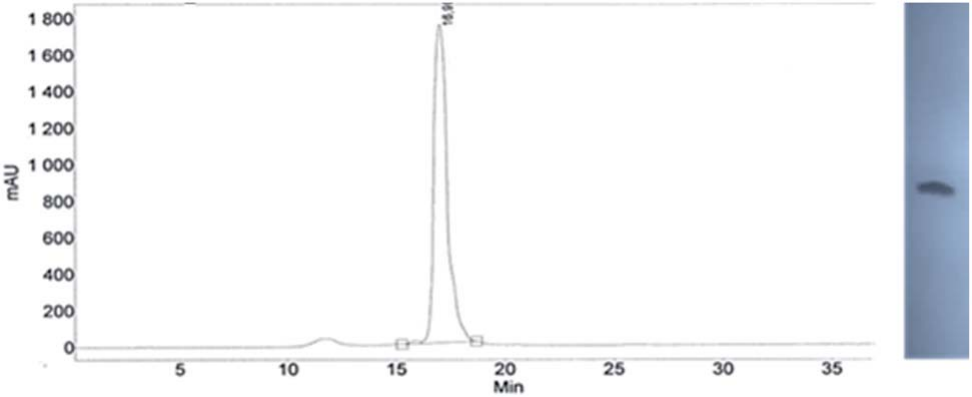
Analysis of 21f obtained from 5Tf. (Left) An RP HPLC profile (DMT-OFF); (right): an electropherogram (20% PAGE).

This modified approach was unsuccessful for the decamers 21 as neither RP nor IE HPLC purification provided products of acceptable purity. Therefore, the DMT-tagged oligomers 21 were isolated by means of RP HPLC using DMT-ON parameters. The final detritylation step was performed using 10% aqueous solution of dichloroacetic acid for 10 minutes, and the final RP HPLC purification was performed. The HPLC profile and the PAGE electropherogram recorded for 21f are shown in Fig. 7, while the relevant ^31^P NMR and MALDI-TOF MS spectra are shown in Fig. S8 (ESI).‡ Unfortunately, because of very low repetitive condensation yields, all attempts to synthesize 21 using 5Ts were unsuccessful. This may indicate that 21m obtained from unresolved 5T predominantly contained the NPS linkages having the P atoms of *R*_P_ absolute configuration, but we were unable to confirm this assumption experimentally. Regretfully, at the time being, the field of antisense applications is inaccessible for uniformly modified P-stereodefined NPS-oligomers, although search for more effective conditions of condensation is going on. However, having resolved the problems with determination of the absolute configurations in the prepared monomers, one can use them in synthesis of precisely tailored probes (*e.g.* for enzymatic studies) bearing P-stereodefined NPS-units in a few preselected positions. As mentioned earlier, elongation of oligomers having at the 5′-end an N_NPS_ unit using an OTP-N monomer is more effective and a PS/NPS chimeric T_PS_dG_NPS_T_PS_dG_NPS_dG oligomer (see observation #4 and Fig. 3, a middle plot) was obtained in 27% overall yield (assessed by the DMT^+^ cation assay). This is an acceptable result when considering the known low efficacy of condensation (see Table 2) of *Pm*-_N_OTP-dG.

Finally, we decided to explore the possibility of making chimeric NPS/PO oligomers. In an attempt to synthesize the 9-mer of the sequence 5′-^DMT^T_PO_(T_PO_)_4_(T_NPS_)_3_T-3′, the NPS-oligo fragment T_NPS_T_NPS_T_NPS_T-$ was assembled using the unresolved 4T, with NEt_3_ added in second and third coupling (Scheme S1, ESI‡). This T_NPS_T_NPS_T_NPS_T-$ core was intended to be elongated with the phosphate units using the phosphoramidite method of DNA synthesis. The standard protocol of the latter method could not be used, because past work in our group on the synthesis of PS/PO chimeras indicated that the anionic internucleotide phosphorothioate linkage is quantitatively converted to the phosphate derivative. This process occurs upon contact with an iodine–base–water solution (which is routinely applied to oxidize the just formed phosphite triester), as well as with many other reagents such as camphorsulfonyloxaziridine,^35^ alkyl hydroperoxides^36^ or peracids.^37^ To a certain extent, this problem was resolved by *S*-alkylation of the PS-oligo core with 2-nitrobenzyl bromide to form the corresponding triester, which can be eventually deprotected with a thiophenolate anion. However, this process has only been optimized for d(N_PS_N) dinucleotides.^38^ Our later studies showed that the destructive PS→PO exchange in PS-oligo is avoided if the P^III^→P^V^ conversion is performed using *t*-Bu-OOSiMe_3_ (ref. 39) (0.33 M in CH_3_CN, 30 min, room temperature).^40^ In the present work, the ^31^P NMR and MALDI-TOF MS measurements revealed that under these conditions the phosphoramidothioate linkage in ^DMT^T_NPS_T_OAc_ also remained unaffected (data not shown). The 9-nt ^DMT^T_PO_(T_PO_)_4_(T_NPS_)_3_T-3′, was obtained in 42% yield as assessed from the DMT^+^ cation assay. Its identity was confirmed by MALDI-TOF MS analysis (Fig. S9, ESI‡). Using 3*H*-1,2-benzodithiol-3-one 1,1-dioxide (a Beaucage reagent), ((dimethylamino-methylidene)amino)-3*H*-1,2,4-dithiazoline-3-thione (DDTT) or phenylacetyl disulfide to sulfurize the P atom in the phosphite triester linkage (formed after the phosphoramidite coupling step) the corresponding PS/NPS chimeras can be obtained.

## Conclusions

In an attempt to synthesize P-stereodefined oligo(deoxyribonucleoside N3′→O5′ phosphoramidothioate)s (NPS-) and chimeric NPS/PO- and NPS/PS-oligomers, 3′-*N*-(2-thio-1,3,2-oxathiaphospholane) derivatives of 5′-*O*-DMT-3′-amino-2′,3′-dideoxy-ribonucleosides (_N_OTP-N), that bear a 4,4-unsubstituted, 4,4-dimethyl, or 4,4-pentamethylene substituted oxathiaphospholane rings, were synthesized. The four monomers 4A, 5C, 5T, and 6G were chromatographically separated into P-diastereomers, which were used in synthesis of dinucleotides N_NPS_N′. One of the intermediate compounds, *i.e.*, ^DMT^dG^iBu^_NPS_T_OAc_, was converted into the *S*-Me derivative, which spontaneously crystallized. Analysis of the relevant diffraction data and the results of stereoselective HINT1 catalyzed hydrolysis of several P-diastereomerically pure N_NPS_N′ provided an evidence that the earlier NMR based assignment of absolute configuration of the P-atom in ^DMT^T_NPSMe_T_OAc_ was incorrect. Mechanistic studies revealed that the relatively low repetitive yield observed during the synthesis of uniformly modified NPS-oligos is caused by a conformational ‘clash’ between adopting a C3′-*endo* conformation the 5′-end nucleoside (3′-amino-2′,3′-dideoxy-, but also 2′-OMe-, or LNA-nucleoside) of a growing oligomer and the incoming _N_OTP-N monomer. This effect is less severe if the 5-end nucleoside (*e.g.* a DNA unit) adopts a C2′-*endo* conformation. Also, an NPS-core can be effectively elongated using the phosphoramidite approach giving rise to chimeric NPS/PO-oligomers.

## Experimental section

### Analytical equipment

^1^H NMR and ^31^P NMR spectra were recorded using Bruker AV-200 (200 MHz for ^1^H) or DRX-500 (500.13 MHz for ^1^H, 125.75 MHz for ^13^C) instruments, with TMS or 85% H_3_PO_4_ used as external standards. High-resolution mass spectra (HRMS) were recorded using a Synapt G2 Si mass spectrometer (Waters) equipped with an ESI source and a quadrupole-time-of-flight mass analyzer. The measurements were performed in negative or positive ion modes, with the capillary and sampling cone voltage set to 2.7 kV and 20 V, respectively. The source temperature was 110 °C. To ensure satisfactory accuracy, data were collected in a centroid mode and the readings were corrected during acquisition using leucine enkephalin as an external reference (Lock-SprayTM), which generated the reference ions at *m*/*z* 554.2615 Da ([M − H]^−^) in the negative ESI mode and at *m*/*z* 556.2771 Da ([M + H]^+^) in a positive ESI mode. The data sets were processed using the MassLynx 4.1 software (Waters). The FAB-MS spectra (13 keV, Cs^+^) were recorded on a Finnigan MAT 95 spectrometer, in positive and negative ion modes. MALDI-TOF MS analyses of oligonucleotides were performed using a Voyager-Elite instrument (PerSeptive Biosystems Inc., Framingham, MA) operating in the reflector mode with the detection of negative ions. All UV absorption measurements were carried out in a 1 cm path-length cell, using a double beam spectrophotometer (CINTRA 10e, GBC, Dandenong, Australia), equipped with a silicon photo-diode detector.

Deprotected dinucleoside (N3′→O5′) phosphoramidothioates were isolated using a binary Varian HPLC system, consisting of two PrepStar 218 pumps and a ProStar 325 UV/VIS detector set at 260 nm. A reverse phase HPLC column (PRP-1, C18, 7 μm, 305 × 7 mm, Hamilton, Reno, NV) was eluted with a gradient of CH_3_CN (1% min^−1^) in 0.1 M TEAB (pH 7.3) at a 2.5 mL min^−1^ flow rate.

Analytical RP HPLC runs were performed using a Kinetex® 5 μm column 100 Å (4.6 × 250 mm, Phenomenex) at a 1 mL min^−1^ flow rate, buffer A, 0.05 M TEAB pH 7.5; buffer B, 40% CH_3_CN in 0.05 M TEAB; a gradient 0 to 40% B over 30 min.

X-ray data were collected on a Bruker APEX III D8 Venture dual microsource system using phi and omega scans with graphite monochromatic Cu Mo Kα (*λ* = 1.54178 Å) radiation.

### Materials

Silica gel chromatography media were supplied by MERCK. TLC silica gel 60 plates were used for routine analyses, while HPTLC silica gel 60 plates were used for the assessment of the chromatographic separability of P-diastereomers of the oxathiaphospholane derivatives; all plates contained a UV F_254_ indicator. Silica gel 60, 200–300 mesh, was used for routine open column chromatographic purification. Protected 3′-amino-2′,3′-dideoxy-ribonucleosides were purchased from Carbosynth Limited (Compton, United Kingdom) or Pharma Waldhof (Germany). 2-Chloro-1,3,2-oxathiaphospholane and its 4,4-substituted analogs were prepared according to the published methods.^4*b*^ Acetonitrile (HPLC grade) used in the syntheses of oligonucleotides was dried over 3 Å molecular sieves until the residual moisture content dropped below 10 ppm (by Karl-Fischer coulometry).

### Phosphitylation/sulfurization of 5′-*O*-DMT-nucleobase-protected 3′-amino-2′,3′-dideoxy-ribonucleosides with 2-chloro-1,3,2-oxathiaphospholane or its 4,4-substituted analogs – a general procedure

To a suspension of 5′-*O*-DMT-nucleobase-protected 3′-amino-2′,3′-dideoxy-nucleosides (1 mmol, the 3′-amino analogs of dA^Bz^, dG^iBu^, T, or dC^Bz^) and elemental sulfur (2 mmol) in dry pyridine (4 mL), appropriate 2-chloro-1,3,2-oxathiaphospholane compound (R = H or Me, or R,R = −(CH_2_)_5_–, 1.2 mmol)^4*b*^ was added dropwise using a gas-tight Hamilton syringe. The mixture was stirred at room temperature until the nucleoside disappeared (*ca.* 3 h, monitored by TLC). The solvent was evaporated and the residue was dissolved in acetonitrile (5 mL). Excess sulfur was filtered off and the filtrate was condensed *in vacuo*. The product (a mixture of P-diastereoisomers) was isolated by “flash” silica gel chromatography (230–400 mesh) using a column eluted with a linear 0→3% gradient of methanol in chloroform containing 0.1% pyridine. The mixtures of P-diastereomers of 4–6 were isolated in good yield (49–88%).

### Separation of the P-diastereomers of _N_OTP-N 4–6

A solution of the appropriate monomer 4–6 in 1.0 mL of the eluent specified in Table 1 was applied to a silica gel column (200 g, 75 × 2 cm). The column was eluted with 300 mL of the eluent and fractions at 10–12 mL were collected and analyzed on HP TLC plates. Yields and ^31^P NMR chemical shifts of the isolated P-epimers are given in Table 1.

### “In solution” synthesis of ^DMT^dG^iBu^_NPSMe_T_OAc_ (10)

To a solution of 5′-*O*-DMT-*N*2-isobutyryl-3′-amino-2′,3′-dideoxy-guanosine-3′-*N*-(2-thio-4,4-pentamethylene-1,3,2-oxathiaphospholane) (6Gf, 0.844 g, 1 mmol) in anhydrous acetonitrile (5 mL), a solution of 3′-*O*-acetyl-thymidine (0.284 g, 1 mmol) and DBU (0.182 g, 1.2 mmol) in anhydrous acetonitrile (3 mL) was added. After 3 h, the reaction mixture was concentrated under reduced pressure. The residue was applied on a silica gel column, which was eluted with a linear 0→30% gradient of methanol in chloroform with 1% of pyridine. The product was isolated in 83% yield (^31^P NMR (CDCl_3_): *δ* 58.53 ppm; FAB MS: *m*/*z* 999.4 (M−1)^−^). To its solution in a mixture of anhydrous acetonitrile (4 mL) and pyridine (1 mL), *N*,*N*-diisopropylethylamine (646 mg, 5 mmol) was added, followed by methyl iodide (710 mg, 5 mmol). The mixture was kept at room temperature overnight and concentrated under reduced pressure. The residue was applied on a silica gel column, which was eluted with a linear 0→10% gradient of methanol in chloroform containing 0.2% of pyridine. The triester was obtained in 63% yield (^31^P NMR (CDCl_3_): *δ* 36.06 ppm; ESI MS: *m*/*z* 1013.32, (M−1)^−^). It crystallized at room temperature from a 50 : 1 (v/v) CHCl_3_/MeOH mixture containing 4% of pyridine.

### Crystallographic analysis of compound 10

Data sets were corrected for Lorentz and polarization effects as well as absorption. The criterion for observed reflections is *I* > 2*σ*(*I*). Lattice parameters were determined from least-squares analysis and reflection data. Empirical absorption corrections were applied using SADABS.^41^ structures were solved by direct methods and refined by full-matrix least-squares analysis on *F*^2^ using X-SEED^42^ equipped with SHELXT.^43^ All non-hydrogen atoms were refined anisotropically by full-matrix least-squares on *F*^2^ using the SHELXL^44^ program. H atoms attached to oxygens were located in difference Fourier maps and refined isotropically with independent O–H distances. The remaining H atoms were included in idealized geometric positions with *U*_iso_ = 1.2*U*_eq_ of the atom to which they were attached (*U*_iso_ = 1.5*U*_eq_ for methyl groups). Molecular configurations were compared to estimated Flack parameters.^45^ The acetate group and one of the methanol solvate molecules exhibit two-part disorder with occupancies refined to 64 : 36 and 53 : 47, respectively. One of the methoxy groups exhibits three-part disorder with occupancies refined to 40 : 37 : 23. These disordered groups were refined with a mixture of restraints to approximate idealized geometries.

### HINT1 catalyzed cleavage of the N_NPS_N′ 11–17

The human Hint1 (HINT1) protein was expressed from the plasmid pSGA02 (ref. 46) in an *E. coli* strain BL 21* and was purified using AMP-agarose (Sigma-Aldrich) affinity chromatography according to the published procedure.^27^ The homogenous enzyme preparations were dialyzed against 20 mM Tris and 150 mM NaCl buffer (pH 7.5), and the resultant solutions were concentrated to a protein concentration 10 mg mL^−1^ and stored at −80 °C.

For the hydrolysis of 11–17, to the 50 μM solutions of the substrates prepared in 20 mM HEPES-Na, 0.5 mM MgCl_2_ buffer (pH 7.2) HINT1 (1–13 μg, with the intention to provide rate of hydrolysis in pmol min^−1^ μg^−1^ protein) was added and the reaction mixtures (of total volume 20 μl) were incubated for 30–120 min at 37 °C. Then, the reaction mixtures were quenched by cooling on ice and analyzed by RP HPLC on a Kinetex column (5 μm C18, 100 Å, 250 × 4.6 mm; Phenomenex) with mobile phases A: 0.05 M TEAB pH 7.5; and B: 40% CH_3_CN in 0.05 M TEAB delivered in a gradient from 0% to 17% B over 15 min, at a flow rate of 1 mL min^−1^. Quantification was performed by integration of peaks of the substrate and products (including desulfured phosphate species) taking into account the number of chromophores in them. As the reference compounds appropriate nucleoside 5′-*O*-phosphorothioates and 5′-*O*-phosphates (dAMPS/dAMP, dGMPS/dGMP, TMPS/TMP) and 3′-amino-2,3′-dideoxy-nucleosides were used. Each experiment was performed in at least triplicate.

### Introduction of N3′→O5′ phosphoramidothioate units during solid-phase synthesis of oligonucleotides

Syntheses were carried out manually using CPG-support, to which a 5′-*O*-DMT-nucleoside unit (1 μmol loaded) was attached with the succinyl-sarcosinyl type linker. A single cycle of chain elongation consisted of the following steps: (1) detritylation [3% dichloroacetic acid in methylene chloride (5 mL); 120 s]; (2) wash [acetonitrile (5 mL), methylene chloride (5 mL)] + drying; (3) coupling [a solution of _N_OTP-N (20 μmol) and DBU (50 μmol) (+50 μmol of NEt_3_ for _N_OTP-T) in 0.15 mL of acetonitrile, freshly premixed; 900 s]; (4) wash [acetonitrile (5 mL), methylene chloride (5 mL)] + drying; (6) capping, [acetic anhydride/DMAP/2,6-lutidine/tetrahydrofuran (0.15 mL), 120 s]; (7) wash [acetonitrile (5 mL), methylene chloride (5 mL)] + drying.

The coupling efficiency was controlled by measurement of the DMT^+^ absorption at 504 nm. The cleavage from the support and nucleobase deprotection were performed with 30% NH_4_OH_aq_ at 55 °C for 8 h. Because post-synthetic detritylation with aqueous acetic acid would destroy the oligomer, after the oligonucleotide chain assembly was completed the “on-instrument” detritylation step was executed. Alternatively, after RP HPLC purification of DMT-tagged oligomers, the DMT group was removed with 10% aqueous solution of dichloroacetic acid for 10 minutes. All oligomers were purified by RP HPLC (a DMT-OFF procedure) and MALDI-TOF MS gave satisfactory results.

### Molecular modeling

All calculations have been performed using a Gaussian 16 (G16) package.^31^ In the calculations aimed at comparison of (*S*_P_)-5′-*O*-DMT-*N*6-benzoyl-2′-deoxyadenosine-3′-*O*-(2-thio-4,4-pentamethylene-1,3,2-oxathiaphospholane) and (*R*_P_)-5′-*O*-DMT-*N*6-benzoyl-3′-amino-2′,3′dideoxy-adenosine-3′-*N*-(2-thio-4,4-pentamethylene-1,3,2-oxathiaphospholane) (presented in Fig. 6), convergence to 10^−8^ in the energy and to 10^−6^ in the density matrix was used. An ultrafine grid with 75 radial shells and 302 angular points was employed. The geometry was optimized without any symmetry restrictions. The geometries of the compounds were generated *de novo* using multiple starting conformations and minimized using the polarizable continuum model (PCM) of the solvent. In two experiments acetonitrile and chloroform were set as the solvent with the standard parameter of dielectric constant *ε* of 35.7 and 4.7, respectively.^47^ Optimizations were performed using the hybrid Hartree–Fock/Density Functional Theory (HF/DFT) method PBE0 (named also as PBE1PBE)^48^ and the 6–311++G** basis set.^49^ Each stationary point was characterized by calculating the harmonic vibration frequencies in order to verify that they have no imaginary frequency. Only the lowest energy structures were taken for the further evaluation.

## Conflicts of interest

There are no conflicts to declare.

## Supplementary Material

RA-010-D0RA04987E-s001

RA-010-D0RA04987E-s002
