# Supplementary material for: P-stereocontrolled synthesis of oligo(nucleoside N3′→O5′ phosphoramidothioate)s – opportunities and limitations
Source: RSC Adv. 2020 Sep 23;10(58):35185–97. doi: 10.1039/d0ra04987e (PMC9056831; doi:10.1039/d0ra04987e)
Supplement: RA-010-D0RA04987E-s001 [file RA-010-D0RA04987E-s001.pdf]

Electronic Supplementary Material (ESI) for RSC Advances.
This journal is © The Royal Society of Chemistry 2020

Supporting Information

P-stereocontrolled synthesis of oligo(nucleoside N3'→O5' phosphoramidothioate)s – opportunities and limitations.

Ewa Radzikowska[a,*], Renata Kaczmarek[a], Dariusz Korczyński[a], Agnieszka Krakowiak[a], Barbara Mikołajczyk[a], Janina Baraniak[a], Piotr Guga[a], Kraig A. Wheeler[b], Tomasz Pawlak[a], and Barbara Nawrot[a]

a) Centre of Molecular and Macromolecular Studies, Polish Academy of Sciences, Sienkiewicza 112. 90-363 Łódź, Poland.
b) Whitworth University, Department of Chemistry, 300 W. Hawthorne Rd., Spokane, WA, 99251 USA.

Table of Content

Data set S1. 5'-*O*-DMT-*N*6-benzoyl-3'-amino-2',3'-dideoxy-adenosine-3'-*N*-(2-thio-1,3,2-oxathiaphospholane) (**4A**). 3

Figure S.1.1. ^{1}H NMR spectrum of 5'-*O*-DMT-*N*6-benzoyl-3'-amino-2',3'-dideoxy-adenosine-3'-*N*-(2-thio-1,3,2-oxathiaphospholane) (**4A**). 4

Figure S.1.2. ^{13}C NMR spectrum of 5'-*O*-DMT-*N*6-benzoyl-3'-amino-2',3'-dideoxy-adenosine-3'-*N*-(2-thio-1,3,2-oxathiaphospholane) (**4A**) 5

Figure S1.3. ^{31}P NMR spectra of the fractions containing P-epimers of 5'-*O*-DMT-*N*6-benzoyl-3'-amino-2',3'-dideoxy-adenosine-3'-*N*-(2-thio-1,3,2-oxathiaphospholane): „*Fast*" **4Af** (an upper panel) and „*Slow*" **4As** + „*Fast*" **4Af** 2:1 (a lower panel). 6

Data Set S2. 5'-*O*-DMT-*N*2-isobutyryl-3'-amino-2',3'-dideoxy-guanosine-3'-*N*-(2-thio-4,4-pentamethylene-1,3,2-oxathiaphospholane) (**6G**). 7

Figure S2.1. ^{1}H NMR spectrum of 5'-*O*-DMT-*N*2-isobutyryl-3-amino-2',3'-dideoxy-guanosine-3'-*N*-(2-thio-4,4-pentamethylene-1,3,2-oxathiaphospholane) (**6G**). 8

Figure S2.2. ^{13}C NMR spectrum of 5'-*O*-DMT-*N*2-isobutyryl-3'-amino-2',3'-dideoxy-guanosine-3'-*N*-(2-thio-4,4-pentamethylene-1,3,2-oxathiaphospholane)) (**6G**). 9

Figure S2.3. ^{31}P NMR spectra of 5'-*O*-DMT-*N*2-isobutyryl-3'-amino-2',3'-dideoxy-guanosine-3'-*N*-(2-thio-4,4-pentamethylene-1,3,2-oxathiaphospholane)) „*Fast*" **6Gf** (upper panel) and „*Slow*" **6Gs** (lower panel). 10

Data set S3. 5'-*O*-DMT-*N*^{4}-benzoyl-3'-amino-2',3'-dideoxy-cytidine-3'-*N*-(2-thio-4,4-dimethyl-1,3,2-oxathiaphospholane) (**5C**). 11

Figure S3.1. ^{1}H NMR spectrum of 5'-*O*-DMT-*N*4-benzoyl-3'-amino-2',3'-dideoxy-cytidine-3'-*N*-(2-thio-4,4-dimethyl-1,3,2-oxathiaphospholane) (**5C**). 12

Figure S3.2. ^{13}C NMR spectrum of 5'-*O*-DMT-*N*4-benzoyl-3'-amino-2',3'-dideoxy-cytidine-3'-*N*-(2-thio-4,4-dimethyl-1,3,2-oxathiaphospholane) (**5C**). 13

Figure S3.3. ^{31}P NMR spectra of 5'-*O*-DMT-*N*4-benzoyl-3'-amino-2',3'-dideoxy-cytidine-3'-*N*-(2-thio-4,4-dimethyl-1,3,2-oxathiaphospholane) „*Fast*" **5Cf** (an upper panel) and „*Slow*" **5Cs** (a lower panel). 14

Data Set S4. 5'-*O*-DMT-3'-amino-3'-deoxy-thymidine-3'-*N*-(2-thio-4,4-dimethyl-1,3,2-oxathiaphospholane) (**5T**). 15

Figure S4.1. ^{1}H NMR spectrum of 5'-*O*-DMT-3'-amino-3'-deoxy-thymidine-3'-*N*-(2-thio-4,4-dimethyl-1,3,2-oxathiaphospholane) (**5T**). 16

Figure S4.2. ^{13}C NMR spectrum of 5'-*O*-DMT-3'-amino-3'-deoxy-thymidine-3'-*N*-(2-thio-4,4-dimethyl-1,3,2-oxathiaphospholane) (**5T**). 17

Figure S4.3. ^{31}P NMR spectra of 5'-*O*-DMT-3'-amino-3'-deoxy-thymidine-3'-*N*-(2-thio-4,4-dimethyl-1,3,2-oxathiaphospholane): „*Fast*" **5Tf** (an upper panel) and „*Slow*" **5Ts** (a lower panel). 18

Figure S5. ^{31}P NMR spectra recorded after synthesis of 5'-*O*-DMT-*N*6-benzoyl-3'-amino-2',3'-dideoxy-adenosine-3'-*N*-(2-thio-1,3,2-oxathiaphospholane). 19

Figure S6.1. A ^{31}P NMR spectrum of $^{DMT}dG^{iBu}{}_{NPSMe}T_{OAc}$ amidodiester (**10f**). 20

Figure S6.2. A ^{1}H NMR spectrum of $^{DMT}dG^{iBu}{}_{NPSMe}T_{OAc}$ amidodiester (**10f**). 21

Figure S6.3. A ^{13}C NMR spectrum of $^{DMT}dG^{iBu}{}_{NPSMe}T_{OAc}$ amidodiester (**10f**). 22

Figure S7. The structure of $^{DMT}dG^{iBu}{}_{NPSMe}T_{OAc}$ amidodiester (**10f**) 23

Figure S8. Analysis of **21** obtained from **5Tf**. Left: a ^{31}P NMR spectrum; right : a MALDI-TOF MS spectrum. 24

Figure S9. A MALDI-TOF MS spectrum recorded for $^{DMT}T_{PO}(T_{PO})_4(T_{NPS})_3T$. 25

Scheme S1. Solid phase synthesis of chimeric NPS/PO oligomer $^{DMT}T_{PO}(T_{PO})_4(T_{NPS})_3T$ 26

Table S1. Isolated yield, HR MS or FAB MS (a negative ions mode), and ^{31}P NMR data for unresolved monomers **4**-**6**. 27

Table S2. Experimental details of crystallographic analysis. 28

Data set S1. 5’-*O*-DMT-*N*6-benzoyl-3’-amino-2’,3’-dideoxy-adenosine-3’-*N*-(2-thio-1,3,2-oxathiaphospholane) (**4A**).

A). HRMS (TOF MS ES+) m/z for $C_{40}H_{40}N_6O_6S_2P$ calculated m/z: 795.2188, found m/z: 795.2191 $[M+H]^+$ and 817.2008 $[M+Na]^+$.

Elemental Composition Report — **Page 1**

Single Mass Analysis
Tolerance = 5.0 PPM / DBE: min = -50.0, max = 80.0
Element prediction: Off
Number of isotope peaks used for i-FIT = 5

Monoisotopic Mass, Even Electron Ions
669 formula(e) evaluated with 8 results within limits (up to 50 closest results for each mass)
Elements Used:
C: 0-43 H: 0-45 N: 0-8 O: 0-8 S: 0-2 P: 0-2
1902018_RK_805A 16 (0.177) Cm (14:19)
TOF MS ES+

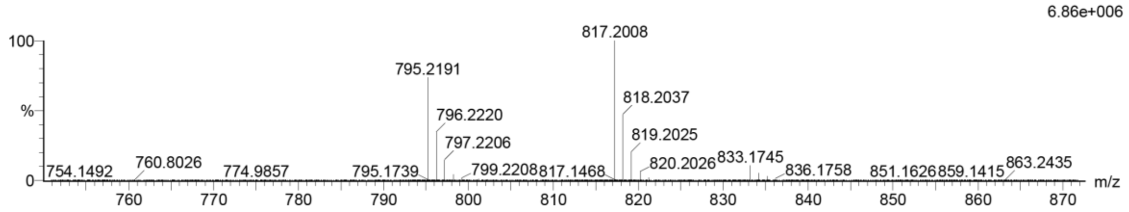

Mass	Calc. Mass	mDa	PPM	DBE	i-FIT	Norm	Conf(%)	Formula
Minimum:				-50.0				
Maximum:		15.0	5.0	80.0				
795.2191	795.2188	0.3	0.4	24.5	1729.5	1.894	15.05	C40 H40 N6 O6 S2 P
	795.2185	0.6	0.8	29.5	1737.0	9.431	0.01	C42 H37 N8 O3 S P2
	795.2205	-1.4	-1.8	19.5	1728.3	0.678	50.77	C38 H45 N4 O7 S2 P2
	795.2172	1.9	2.4	29.5	1732.8	5.226	0.54	C42 H35 N8 O5 S2
	795.2210	-1.9	-2.4	25.5	1749.6	22.055	0.00	C38 H37 N8 O8 P2
	795.2171	2.0	2.5	24.5	1736.9	9.280	0.01	C41 H41 N4 O7 S P2
	795.2218	-2.7	-3.4	24.5	1728.7	1.090	33.62	C39 H41 N8 O3 S2 P2
	795.2155	3.6	4.5	29.5	1736.7	9.081	0.01	C43 H36 N6 O6 S P

B) ^1H NMR ($CDCl_3$, δ, ppm): 8.66 (d, *J*=11.4 Hz, 1H, H-2), 8.19 (d, *J*=21.0 Hz, 1H, H-8), 7.95 (br.d, *J*=7.6 Hz, 2H, Ph), 7.50-7.45 (m, 1H, Ph), 7.41-7.35 (m, 2H, Ph), 7.33-7.27 (m, 2H, Ph), 7.22-7.11 (m, 7H, Ph), 7.10-7.05 (m, 1H, Ph), 6.68 (d, *J*=8.8 Hz, 3H, Ph), 6.36-6.31 (m, 1H, H-1’), 5.82-5.73 (m, 1H, H-3’), 4.53-4.45 (m, 1H, H-4’), 4.11-4.00 (m, 2H, H-5’, H-5”), 3.64 (br.s, 6H, 2xCH_3 from DMT group), 3.62-3.58 (m, 1H, H-2’), 3.46-3.42 (m, 1H, H-2”), 3.36-3.28 (m, 2H, CH_2-OTP), 3.26-3.18 (m, 1H, CH_2-OTP), 3.09-2.95 (m, 1H, CH_2-OTP).

^{13}C NMR ($CDCl_3$, δ, ppm): 158.53; 152.35; 151.18; 151.13; 149.81; 144.59; 144.48; 141.97; 141.78; 136.00; 135.70; 135.67; 135.60; 135.56; 135.50; 135.31; 133.74; 132.76; 130.05; 128.11; 126.92; 123.76; 113.13; 86.58; 86.50; 85.03; 84.98; 84.53; 84.22; 68.68; 68.58; 63.12; 62.56; 55.23; 53.46; 52.83; 39.69; 39.18; 37.24; 37.17.

C) The P-epimers were partially separated into two fractions: the nearly pure „*Fast*”- and „*Slow*”-eluting-enriched (**4Af**:**4As,** 1:2). Chromatography on a silica gel 60H column was performed using a 0→1% gradient of methanol in chloroform with 0.2% addition of pyridine as the eluent.

„*Fast*”-**4Af**: isolated yield 12%;

^{31}P NMR ($CDCl_3$) δ: 95.92 ppm;

„*Slow*”-**4As**: + „*Fast*”-**4Af** (2:1) isolated yield 24%;

^{31}P NMR ($CDCl_3$) δ: 95.25 ppm, 96.04 ppm, respectively.

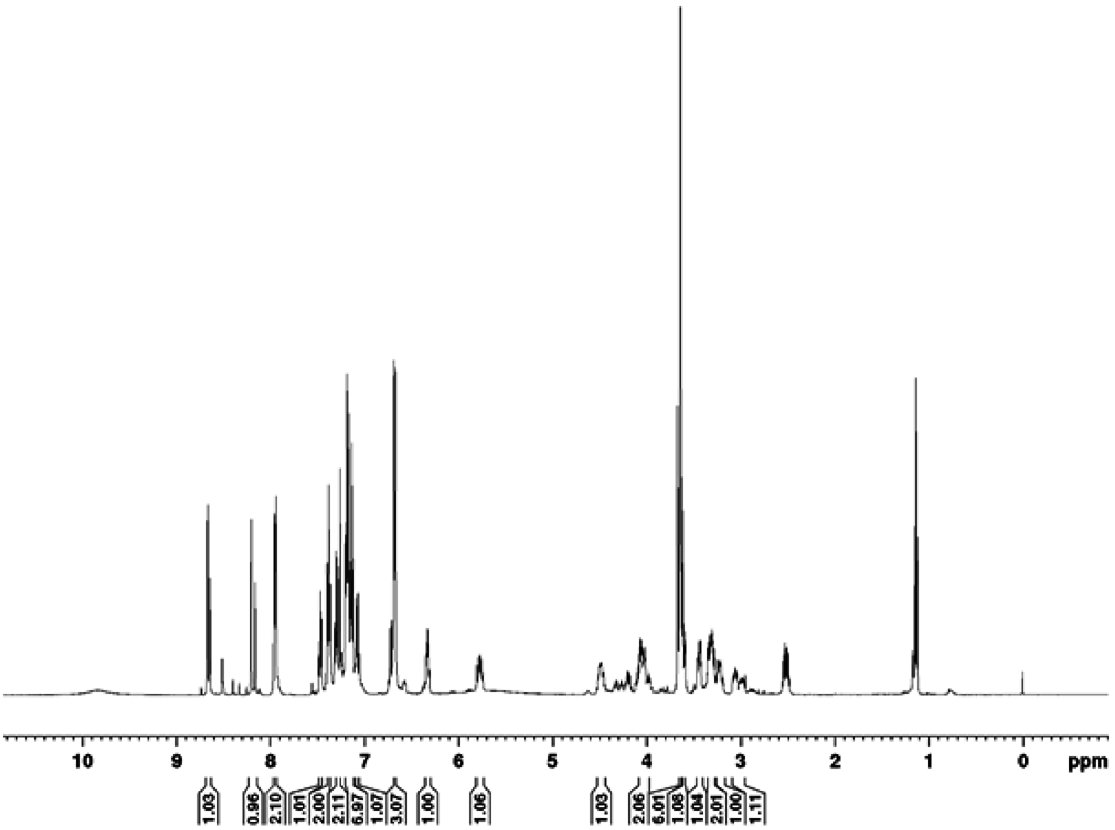

Figure S.1.1. ^{1}H NMR spectrum of 5’-*O*-DMT-*N*6-benzoyl-3’-amino-2’,3’-dideoxy-adenosine-3’-*N*-(2-thio-1,3,2-oxathiaphospholane) (**4A**).

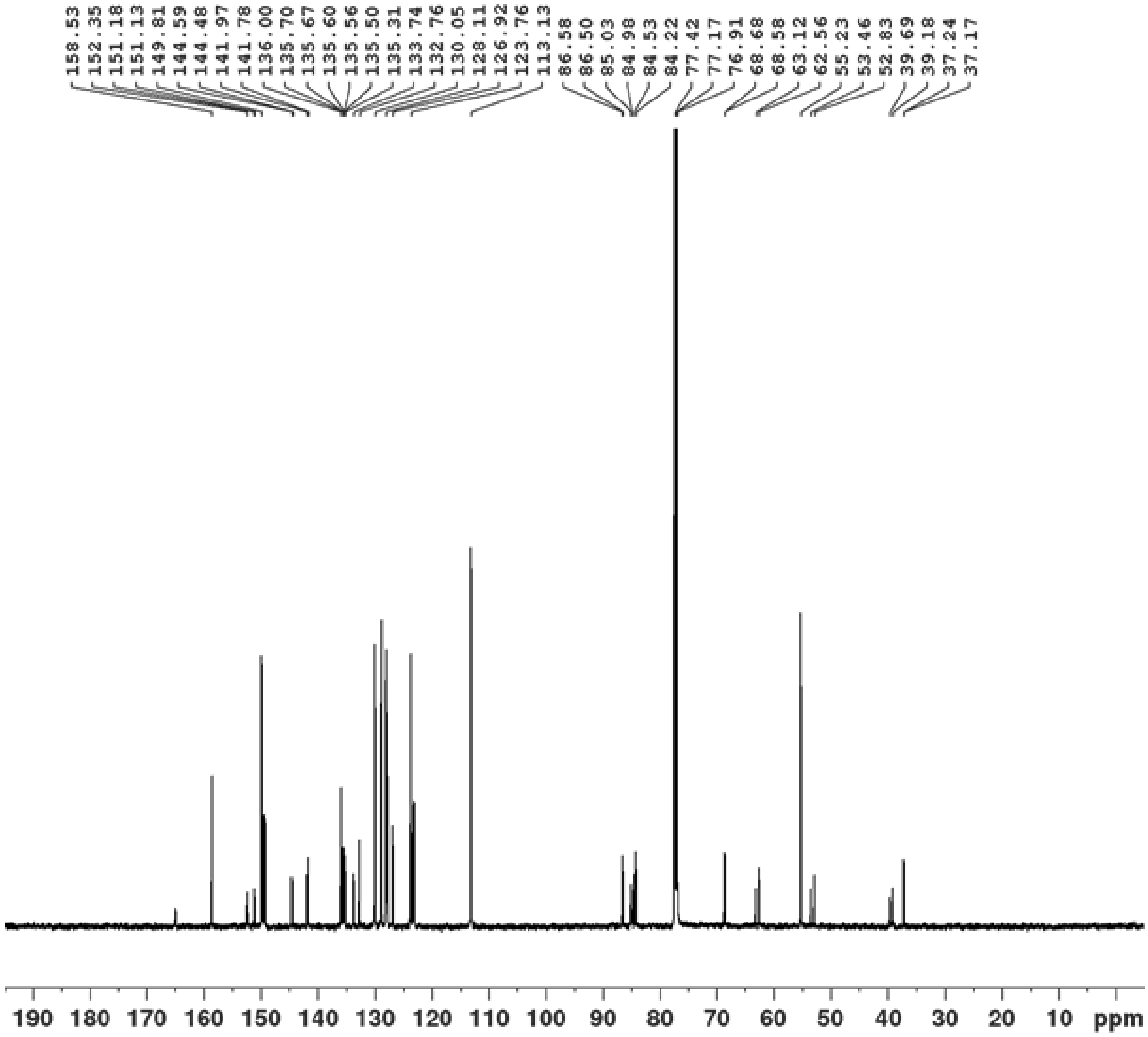

Figure S.1.2. ^{13}C NMR spectrum of 5’-*O*-DMT-*N*6-benzoyl-3’-amino-2’,3’-dideoxy-adenosine-3’-*N*-(2-thio-1,3,2-oxathiaphospholane) (**4A**)

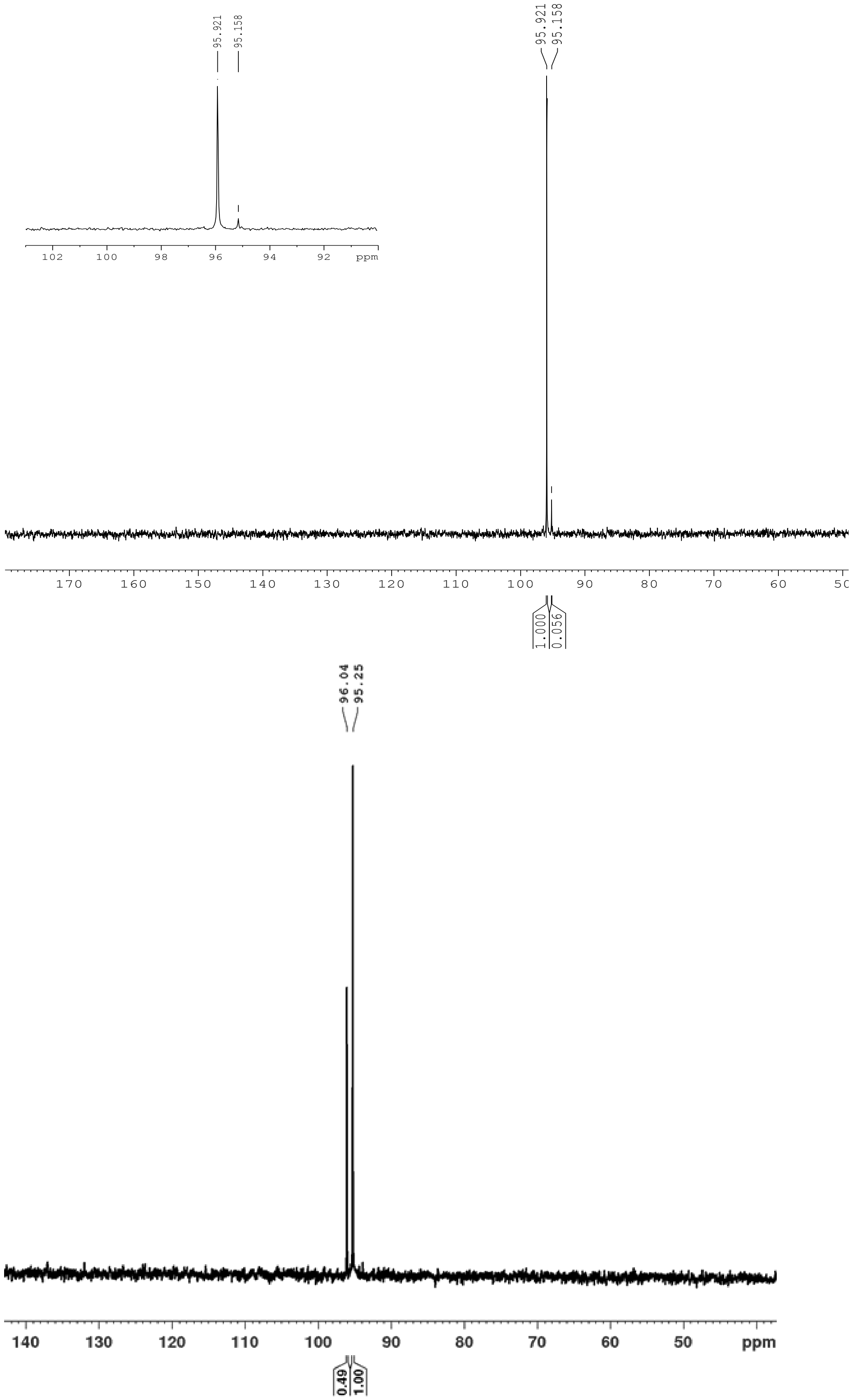

Figure S1.3. ^{31}P NMR spectra of the fractions containing P-epimers of 5'-*O*-DMT-*N*6-benzoyl-3'-amino-2',3'-dideoxy-adenosine-3'-*N*-(2-thio-1,3,2-oxathiaphospholane): „*Fast*" **4Af** (an upper panel) and „*Slow*" **4As** + „*Fast*" **4Af** 2:1 (a lower panel).

Data Set S2. 5'-*O*-DMT-*N*2-isobutyryl-3'-amino-2',3'-dideoxy-guanosine-3'-*N*-(2-thio-4,4-pentamethylene-1,3,2-oxathiaphospholane) (**6G**).

A) HRMS (TOF MS ES+) m/z for $C_{42}H_{50}N_6O_7S_2P$ calculated m/z: 845.2920, found m/z: 845.2920 $[M+H]^+$ and 867.2737 $[M+Na]^+$.

Elemental Composition Report **Page 1**

Single Mass Analysis
Tolerance = 5.0 PPM / DBE: min = -50.0, max = 80.0
Element prediction: Off
Number of isotope peaks used for i-FIT = 5

Monoisotopic Mass, Even Electron Ions
551 formula(e) evaluated with 4 results within limits (up to 50 closest results for each mass)
Elements Used:
C: 0-45 H: 0-50 N: 0-8 O: 0-8 S: 0-2 P: 0-2

1902018_RK_4BA 16 (0.177) Cm (13:17-(33:72+2:8))
TOF MS ES+

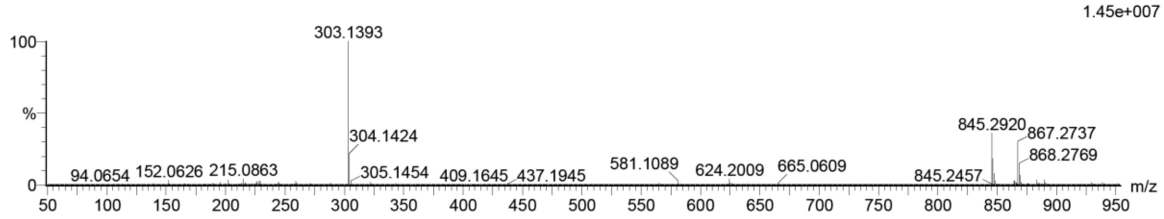

Minimum:				-50.0				
Maximum:		15.0	5.0	80.0				
Mass	Calc. Mass	mDa	PPM	DBE	i-FIT	Norm	Conf(%)	Formula
845.2920	845.2920	0.0	0.0	21.5	1797.1	0.024	97.67	C42 H50 N6 O7 S2 P
	845.2916	0.4	0.5	26.5	1808.2	11.102	0.00	C44 H47 N8 O4 S P2
	845.2903	1.7	2.0	26.5	1800.8	3.762	2.32	C44 H45 N8 O6 S2
	845.2886	3.4	4.0	26.5	1807.7	10.640	0.00	C45 H46 N6 O7 S P

B) ^{1}H NMR ($CDCl_3$, δ, ppm): 11.99 (br.s, 1H, -NH), 11.81 (s, 1H, -NH), 8.27 (d, *J*=6.7 Hz, 1H, N2-H), 7.71 (s, 1H, H-8), 7.08 (d, *J*=7.7 Hz, 2H, Ph), 6.99-6.95 (m, 4H, Ph), 5.89-5.84 (m, 1H, H-1'), 6.85-6.80 (m, 1H, Ph), 6.51-6.44 (m, 4H, Ph), 4.34-4.23 (m, 1H, H3'), 3.89-3.85 (m, 1H, H-4'), 3.72-3.65 (m, 1H, H-5'), 3.61-3.51 (m, 1H, H-5"), 3.38 (s, 3H, CH_3 from DMT group), 3.37 (s, 3H, CH_3 from DMT group), 3.21-3.17 (m, 1H, H-2'), 3.05-3.01 (m, 1H, H-2"), 2.60-2.45 (m, 2H, CH_2-OTP), 2.26-2.17 (m, 1H, CH from *i*-Bu), 1.76-1.70 (m, 1H, CH_2-OTP), 1.53-1.46 (m, 1H, CH_2-OTP), 1.33-1.08 (m, 4H, CH_2-OTP); 1.06—0.97 (m, 2H, CH_2-OTP), 0.96 (s, 3H, CH_3 from *i*-Bu), 0.94 (s, 3H, from *i*-Bu), 0.87-0.77 (m, 2H CH_2-OTP);

^{13}C NMR ($CDCl_3$, δ, ppm): 179.51; 158.50; 155.76; 149.68; 147.87; 144.58; 139.91; 137.67; 136.16; 135.86; 135.66; 135.62; 135.47; 130.09; 129.24; 128.13; 127.91; 127.82; 127.74; 126.86; 123.84; 121.53; 113.11; 113.05; 86.45; 84.78; 84.71; 83.97; 81.22; 67.95; 63.14; 55.22; 53.35; 39.54; 37.37; 37.13; 36.17; 25.21; 23.82; 23.64; 19.02; 19.00.

C) The P-epimers were separated on a silica gel column using chloroform : methanol (50:1; v/v) mixture (with 0.2% of pyridine) as an eluent.

„*Fast*"-**6Gf**: isolated yield 46%;

^{31}P NMR ($CDCl_3$) δ: 96.92 ppm;

„*Slow*"-**6Gs**: isolated yield 32%;

^{31}P NMR ($CDCl_3$) δ: 96.94 ppm;

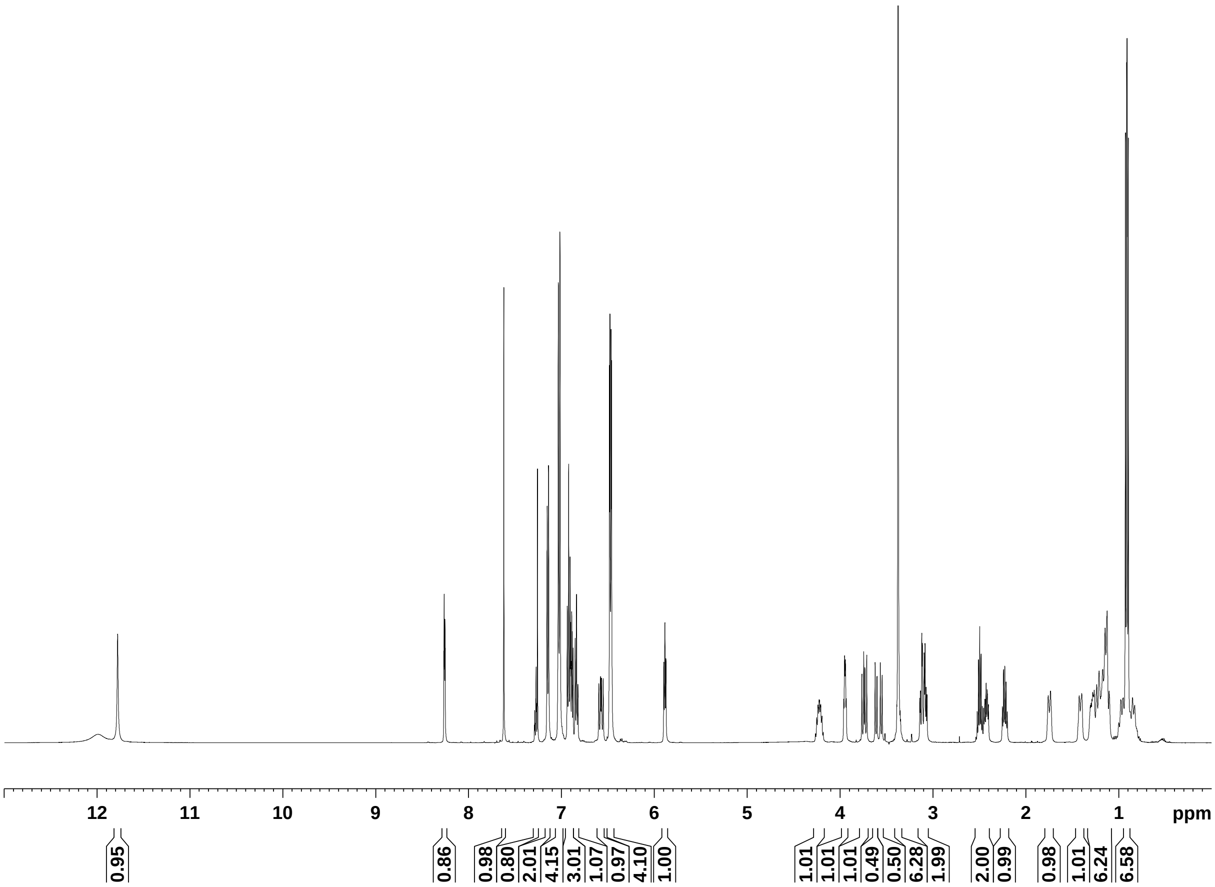

Figure S2.1. ^{1}H NMR spectrum of 5’-*O*-DMT-*N*2-isobutyryl-3-amino-2’,3’-dideoxy-guanosine-3’-*N*-(2-thio-4,4-pentamethylene-1,3,2-oxathiaphospholane) (**6G**).

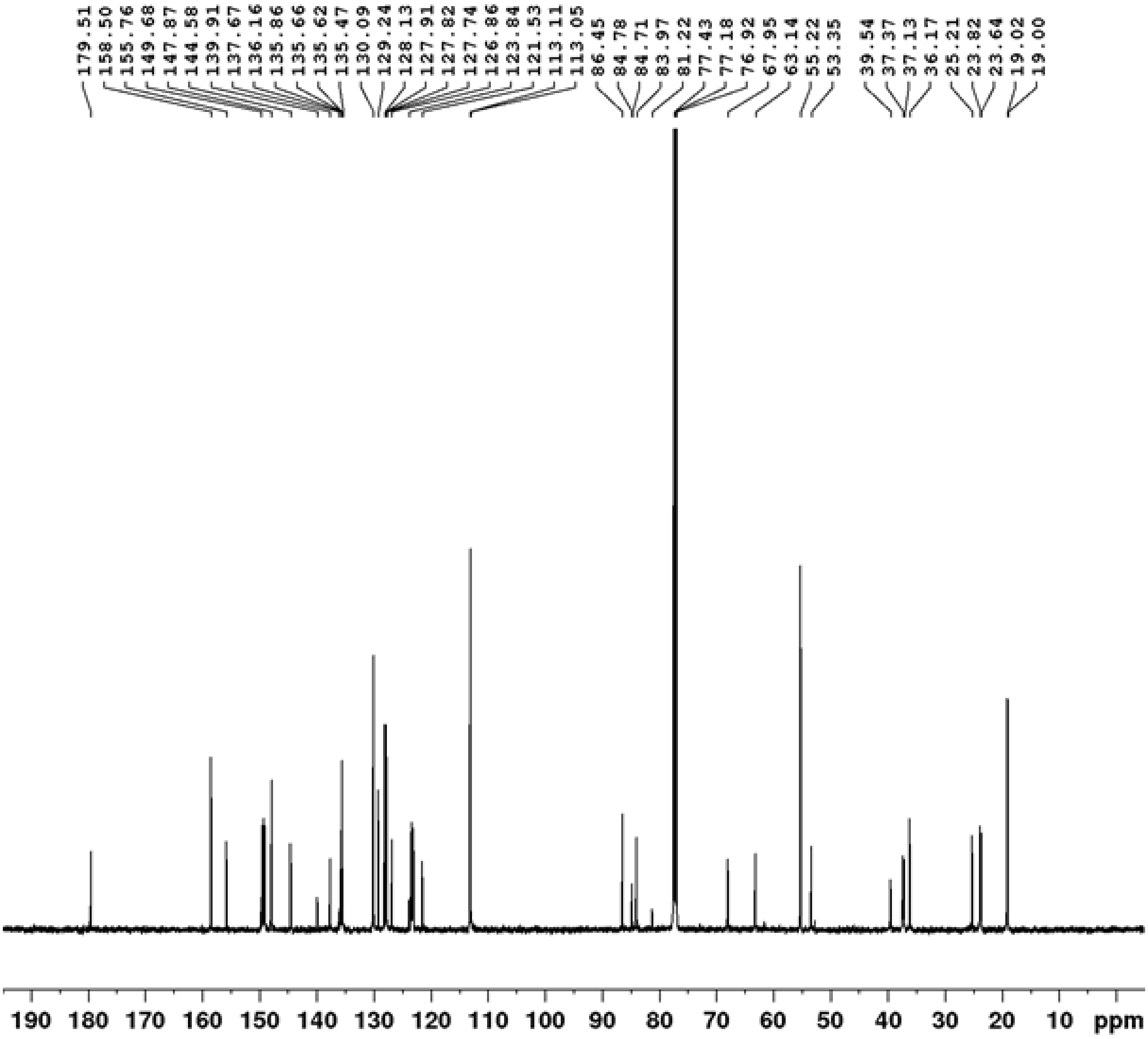

Figure S2.2. ^{13}C NMR spectrum of 5’-*O*-DMT-*N*2-isobutyryl-3’-amino-2’,3’-dideoxy-guanosine-3’-*N*-(2-thio-4,4-pentamethylene-1,3,2-oxathiaphospholane)) (**6G**).

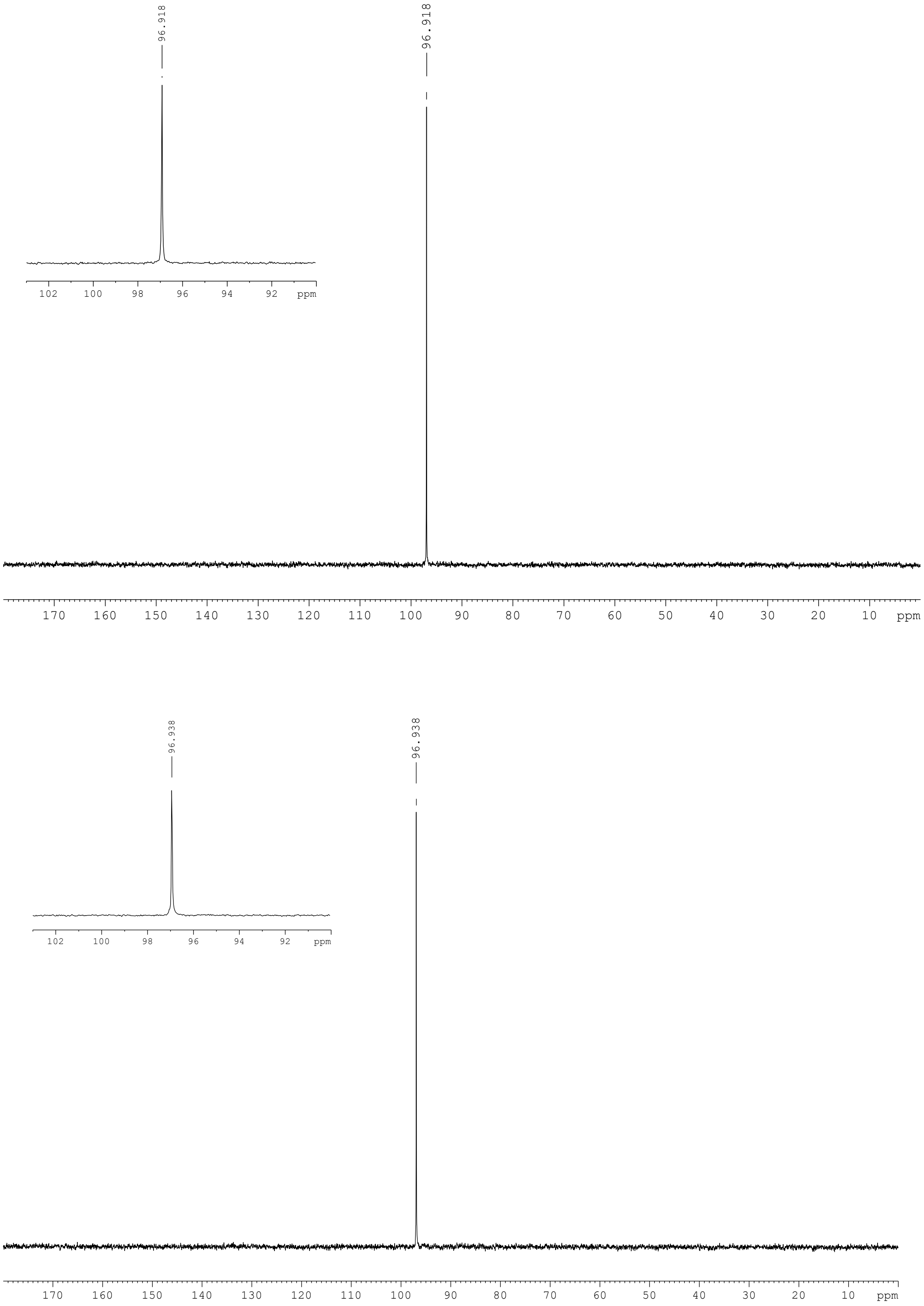

Figure S2.3. ^{31}P NMR spectra of 5'-*O*-DMT-*N*2-isobutyryl-3'-amino-2',3'-dideoxy-guanosine-3'-*N*-(2-thio-4,4-pentamethylene-1,3,2-oxathiaphospholane)) „*Fast*" **6Gf** (upper panel) and „*Slow*" **6Gs** (lower panel).

Data set S3. 5'-*O*-DMT-*N*^{4}-benzoyl-3'-amino-2',3'-dideoxy-cytidine-3'-*N*-(2-thio-4,4-dimethyl-1,3,2-oxathiaphospholane) (**5C**).

A). HRMS (TOF MS ES+) m/z for $C_{41}H_{44}N_4O_7S_2P$ calculated m/z: 799.2389, found m/z: 799.2385, $[M+H]^+$ and 821.2198 $[M+Na]^+$.

Elemental Composition Report **Page 1**

Single Mass Analysis
Tolerance = 5.0 PPM / DBE: min = -50.0, max = 80.0
Element prediction: Off
Number of isotope peaks used for i-FIT = 5

Monoisotopic Mass, Even Electron Ions
323 formula(e) evaluated with 2 results within limits (up to 50 closest results for each mass)
Elements Used:
C: 0-43 H: 0-45 N: 0-5 O: 0-8 S: 0-2 P: 0-2
1902018_RK_843A 13 (0.151) Cm (13:22)
TOF MS ES+

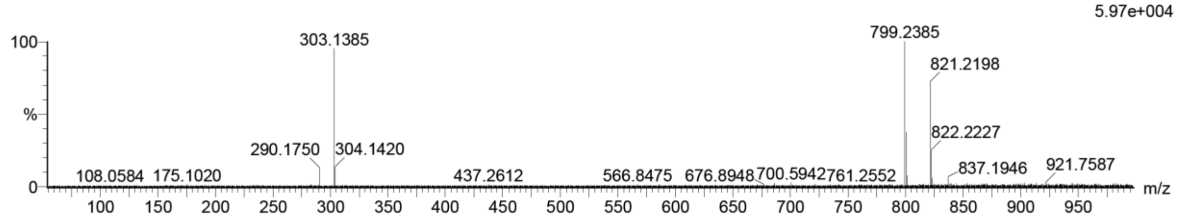

Minimum:				-50.0				
Maximum:		15.0	5.0	80.0				

Mass	Calc. Mass	mDa	PPM	DBE	i-FIT	Norm	Conf(%)	Formula
799.2385	799.2389	-0.4	-0.5	22.5	1226.7	0.450	63.75	C41 H44 N4 O7 S2 P
	799.2372	1.3	1.6	22.5	1227.3	1.015	36.25	C42 H45 N2 O8 S P2

B). ^{1}H NMR ($CDCl_3$, δ, ppm): 8.40 (d, *J*=8.1 Hz, 1H, H-6), 7.93 (d, *J*=8.1 Hz, 2H, Ph), 7.64-7.44 (m, 5H, H-5, Ph), 7.39-7.29 (m, 6H, Ph), 7.28-7.23 (m, 2H, Ph), 6.94-6.83 (m, 4H, Ph), 6.12-6.04 (m, 1H, H-1'), 4.73-4.59 (m, 1H, H-3'), 4.47-4.29 (m, 1H, H-4'), 4.08-3.92 (m, 2H, CH_2-OTP), 3.76 (br.s, 6H, 2xCH_3 from DMT group), 3.45-3.40 (m, 2H, H-5', H-5"), 2.50-2.39 (m, 2H, H-2', H-2"), 1.54 (s, 3H, CH_3-OTP), 1.46 (s, 3H, CH_3-OTP);

^{13}C NMR ($CDCl_3$, δ, ppm): 162.37; 158.70; 149.74; 144.82; 144.16; 144.11; 136.11; 135.50; 135.26; 135.20; 133.18; 133.08; 130.30; 130.16; 128.96; 127.21; 127.17; 123.82; 113.35; 87.19; 87.09; 86.62; 86.34; 85.29; 85.21; 85.03; 84.97; 78.73; 78.62; 61.34; 61.14; 61.02; 55.27; 51.61; 51.38; 41.42; 40.94; 28.77; 28.55; 28.45; 28.23.

C) The P-epimers were separated on a silica gel column using a gradient of methanol in chloroform (0→2%) with 0.2% of pyridine as the eluent.

„*Fast*"-**5Cf**: isolated yield 13%;

^{31}P NMR ($CDCl_3$) δ: 97.74 ppm.

„*Slow*"-**5Cs**: isolated yield 32%;

^{31}P NMR ($CDCl_3$) δ: 97.25 ppm.

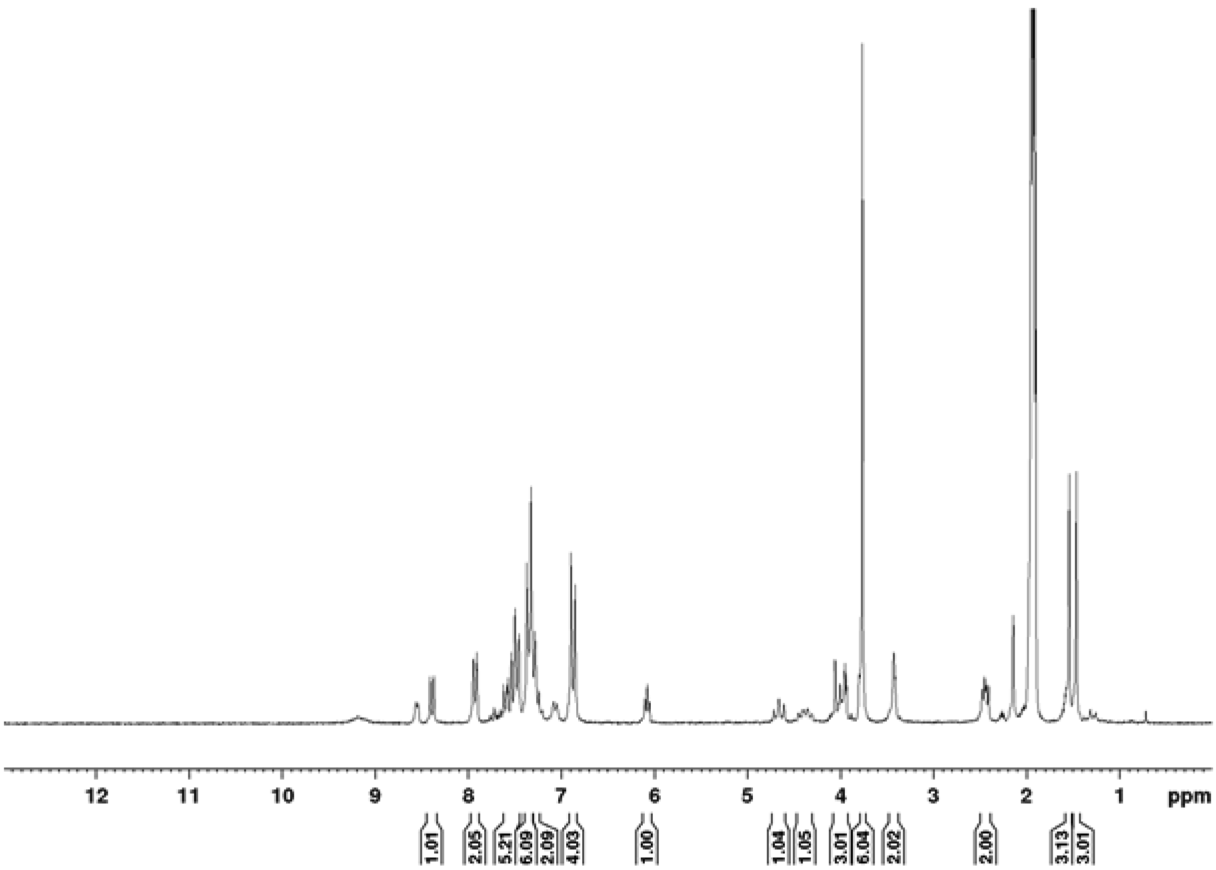

Figure S3.1. ^{1}H NMR spectrum of 5’-*O*-DMT-*N*4-benzoyl-3’-amino-2’,3’-dideoxy-cytidine-3’-*N*-(2-thio-4,4-dimethyl-1,3,2-oxathiaphospholane) (**5C**).

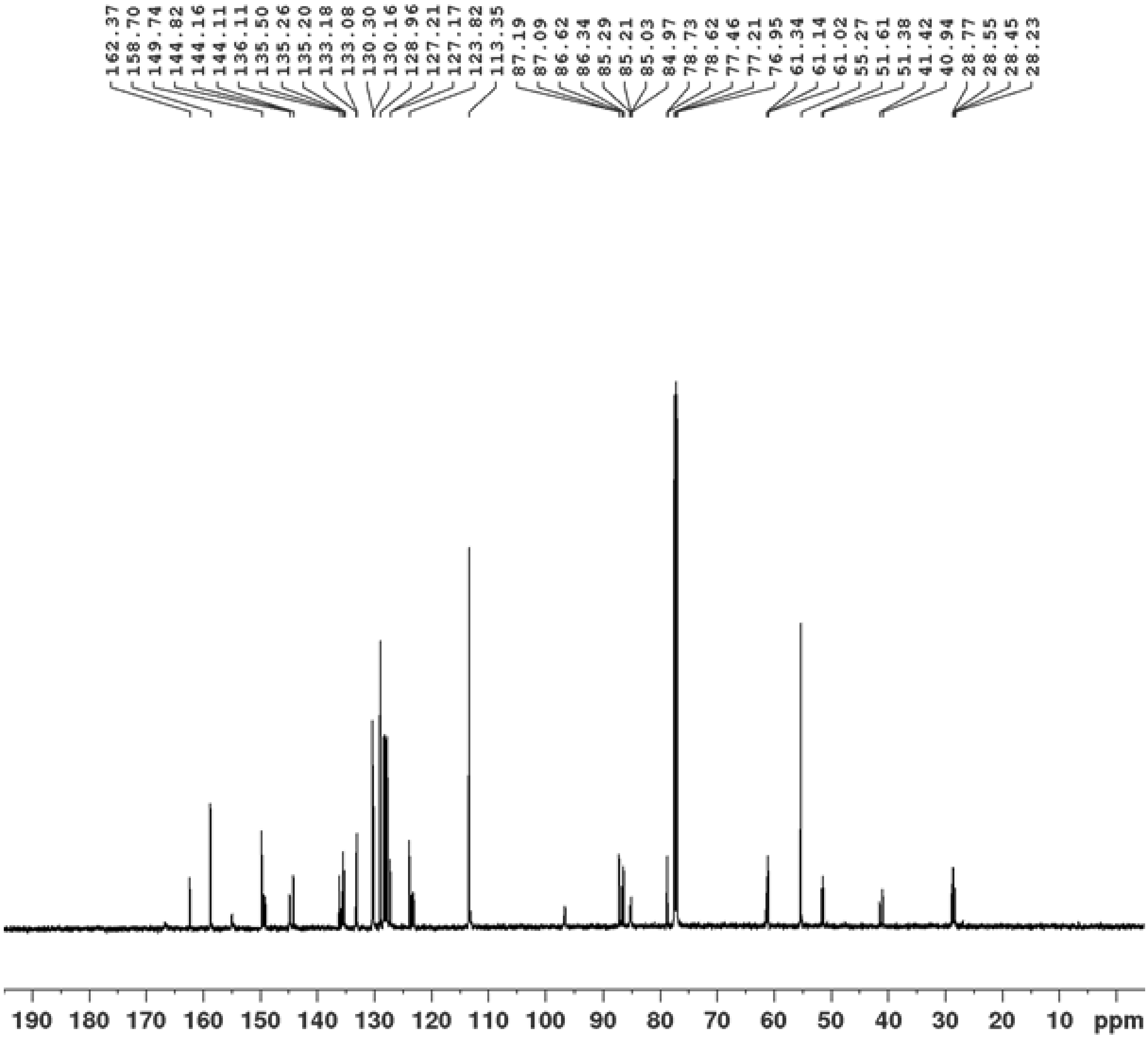

Figure S3.2. ^{13}C NMR spectrum of 5’-*O*-DMT-*N*4-benzoyl-3’-amino-2’,3’-dideoxy-cytidine-3’-*N*-(2-thio-4,4-dimethyl-1,3,2-oxathiaphospholane) (**5C**).

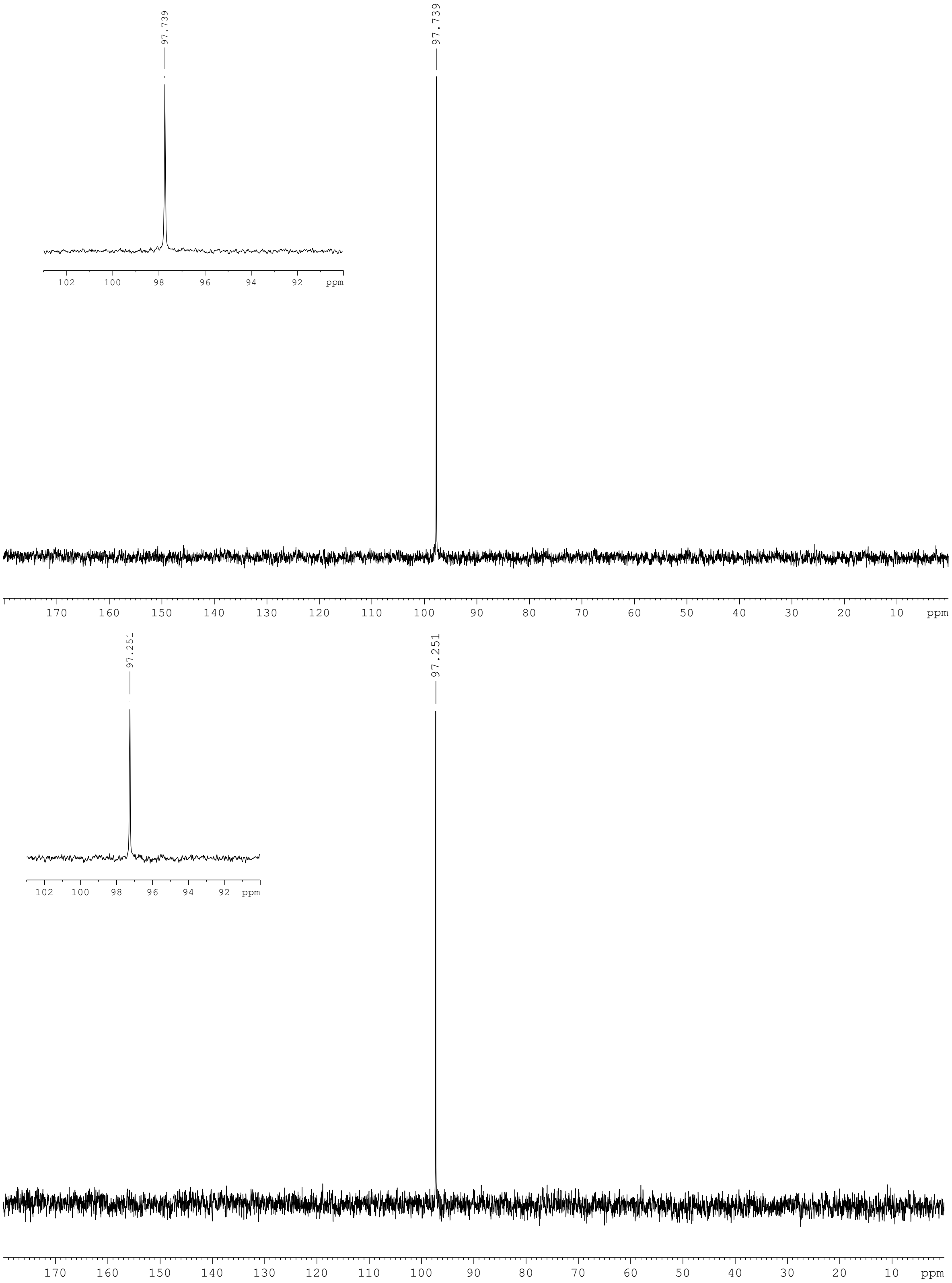

Figure S3.3. ^{31}P NMR spectra of 5'-*O*-DMT-*N*4-benzoyl-3'-amino-2',3'-dideoxy-cytidine-3'-*N*-(2-thio-4,4-dimethyl-1,3,2-oxathiaphospholane) „*Fast"* **5Cf** (an upper panel) and „*Slow*" **5Cs** (a lower panel).

Data Set S4. 5’-*O*-DMT-3’-amino-3’-deoxy-thymidine-3’-*N*-(2-thio-4,4-dimethyl-1,3,2-oxathiaphospholane) (**5T**).

A) HRMS (TOF MS ES+) m/z for $C_{35}H_{40}N_3O_7S_2PNa$ calculated m/z: 732.1943, found m/z: 732.1951 $[M+Na]^+$.

Elemental Composition Report **Page 1**

Single Mass Analysis
Tolerance = 5.0 PPM / DBE: min = -50.0, max = 80.0
Element prediction: Off
Number of isotope peaks used for i-FIT = 5

Monoisotopic Mass, Even Electron Ions
602 formula(e) evaluated with 7 results within limits (up to 50 closest results for each mass)
Elements Used:
C: 0-40 H: 0-45 N: 0-5 O: 0-8 S: 0-2 P: 0-2 Na: 1-1

1902018_RK_823A 16 (0.177) Cm (13:18)
TOF MS ES+

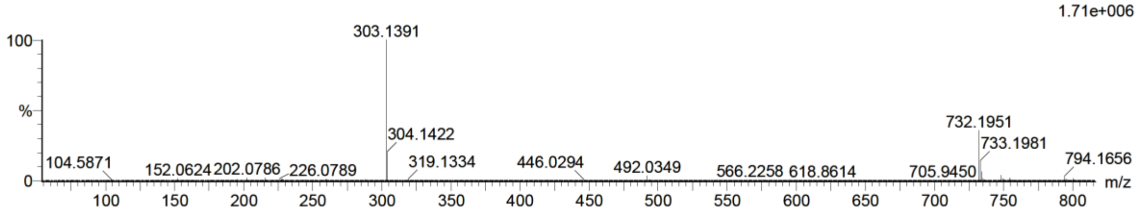

Minimum:				-50.0				
Maximum:		15.0	5.0	80.0				
Mass	Calc. Mass	mDa	PPM	DBE	i-FIT	Norm	Conf(%)	Formula
732.1951	732.1943	0.8	1.1	17.5	804.0	2.610	7.36	C35 H40 N3 O7 S2 P Na
	732.1960	-0.9	-1.2	12.5	802.7	1.397	24.72	C33 H45 N O8 S2 P2 Na
	732.1939	1.2	1.6	22.5	802.6	1.281	27.79	C37 H37 N5 O4 S P2 Na
	732.1973	-2.2	-3.0	17.5	803.4	2.080	12.50	C34 H41 N5 O4 S2 P2 Na
	732.1926	2.5	3.4	22.5	806.2	4.890	0.75	C37 H35 N5 O6 S2 Na
	732.1926	2.5	3.4	17.5	802.7	1.323	26.62	C36 H41 N O8 S P2 Na
	732.1983	-3.2	-4.4	21.5	807.3	5.944	0.26	C40 H40 N O5 S2 P Na

B) ^{1}H NMR ($CDCl_3$, δ, ppm): 11.45 (br.s, 1H, -NH), 7.55-7.23 (m, 3H, H-6, Ph), 7.28-7.14 (m, 7H, Ph), 6.79-6.69 (m, 4H, Ph) 6.12-5.95 (m, 1H, H-1’), 4.53-4.41 (m, 1H, H-3’), 4.03-3.87 (m, 2H, CH_2-OTP), 3.81-3.72 (m, 1H, H-4’), 3.66 (br.s, 6H, 2xCH_3 from DMT group), 3.43-3.28 (m, 2H, H-5’, H-5”), 2.46-2.32 (m, 2H, H-2’, H-2”), 1.52-1.24 (m, 9H, 3xCH_3 from C5 and OTP);

^{13}C NMR ($CDCl_3$, δ, ppm): 164.41; 158.63; 151.01; 149.49; 149.28; 149.06; 144.43; 135.66; 135.47; 135.27; 133.15; 130.18; 130.14; 128.19; 127.92; 127.02; 123.71; 123.36; 123.16; 122.96; 113.21; 111.26; 86.78; 85.27; 85.21; 84.32; 78.42; 62.87; 60.82; 55.16; 53.16; 40.42; 28.51; 28.22; 11.83.

C) The P-epimers were separated on a silica gel column using ethyl acetate : hexane (1:1, v/v) (no pyridine added) as an eluent.

„*Fast*”-**5Tf**: isolated yield 29%;

^{31}P NMR ($CDCl_3$) δ: 96.69 ppm;

„*Slow*”-**5Ts**: isolated yield 15%;

^{31}P NMR ($CDCl_3$) δ: 96.58 ppm.

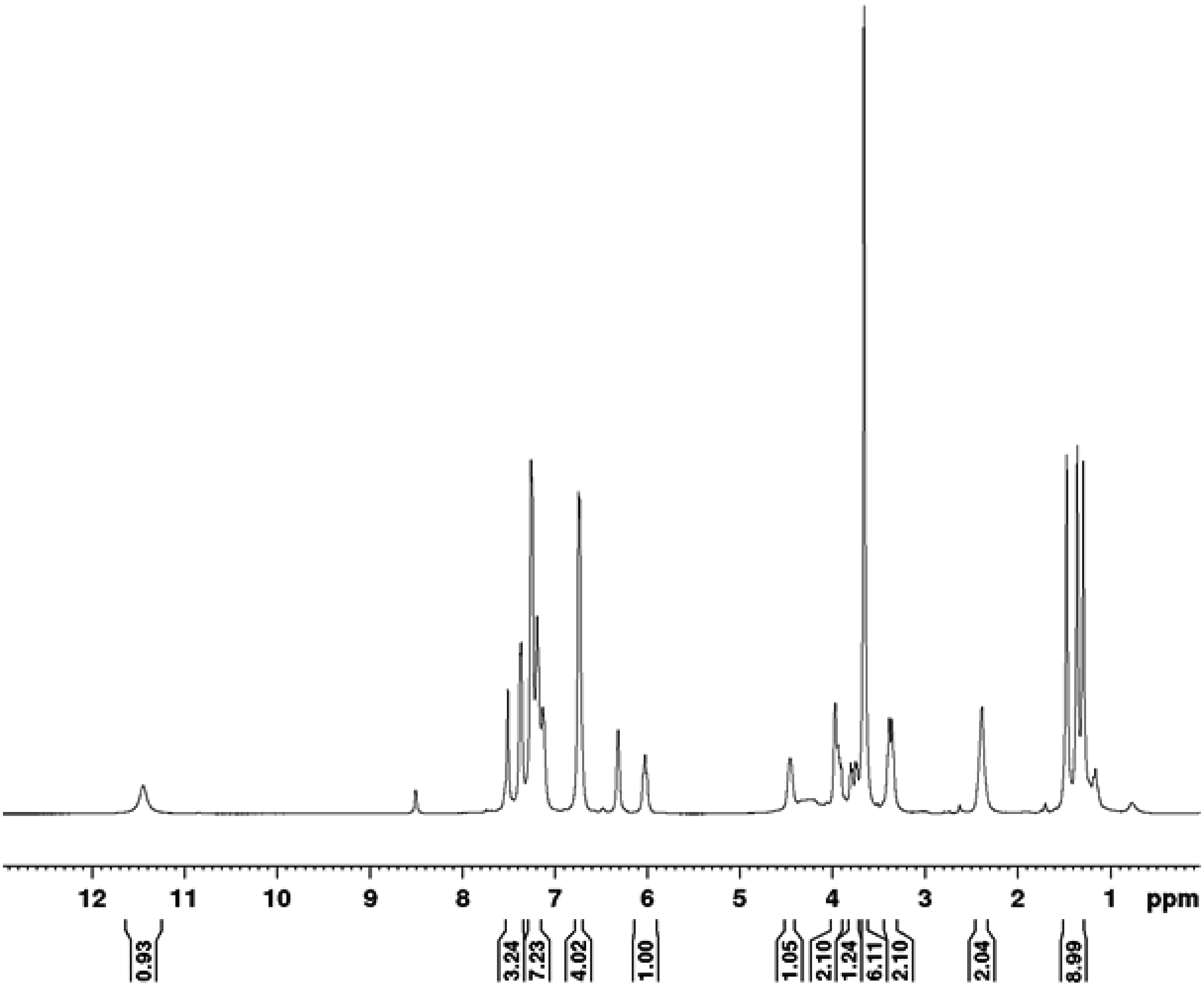

Figure S4.1. ^{1}H NMR spectrum of 5’-*O*-DMT-3’-amino-3’-deoxy-thymidine-3’-*N*-(2-thio-4,4-dimethyl-1,3,2-oxathiaphospholane) (**5T**).

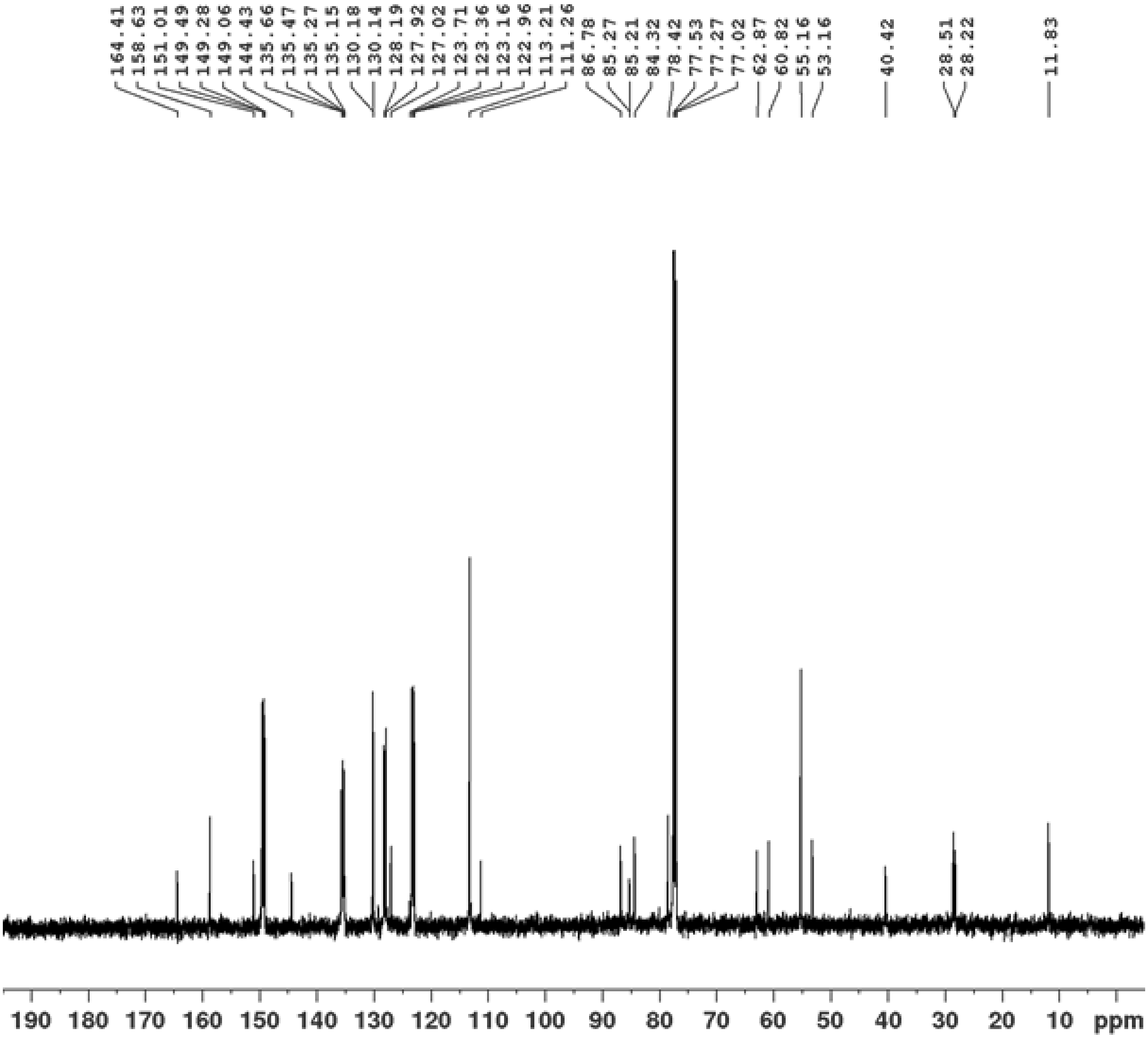

Figure S4.2. ^{13}C NMR spectrum of 5’-*O*-DMT-3’-amino-3’-deoxy-thymidine-3’-*N*-(2-thio-4,4-dimethyl-1,3,2-oxathiaphospholane) (**5T**).

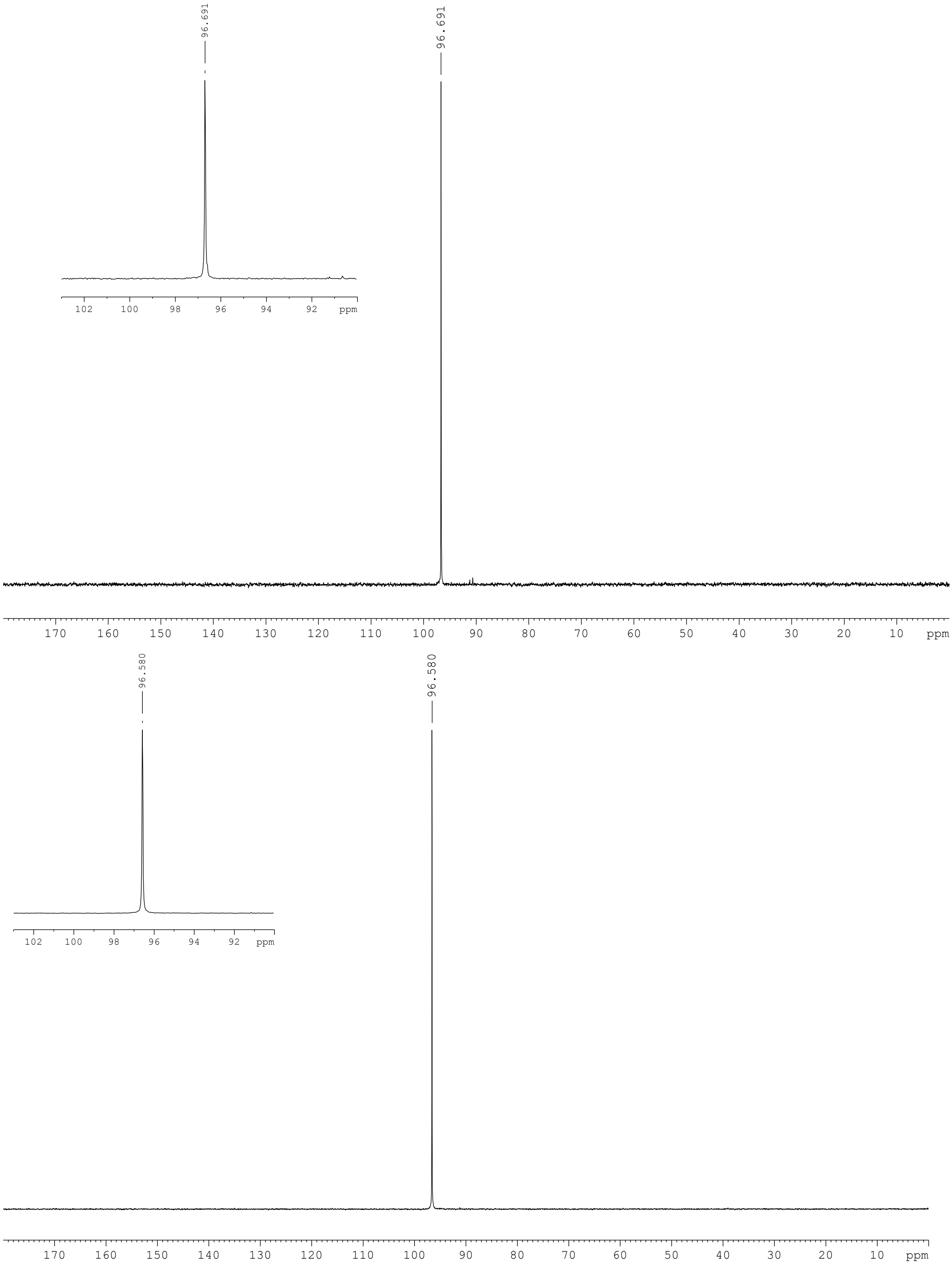

Figure S4.3. ^{31}P NMR spectra of 5’-*O*-DMT-3’-amino-3’-deoxy-thymidine-3’-*N*-(2-thio-4,4-dimethyl-1,3,2-oxathiaphospholane): „*Fast*” **5Tf** (an upper panel) and „*Slow*” **5Ts** (a lower panel).

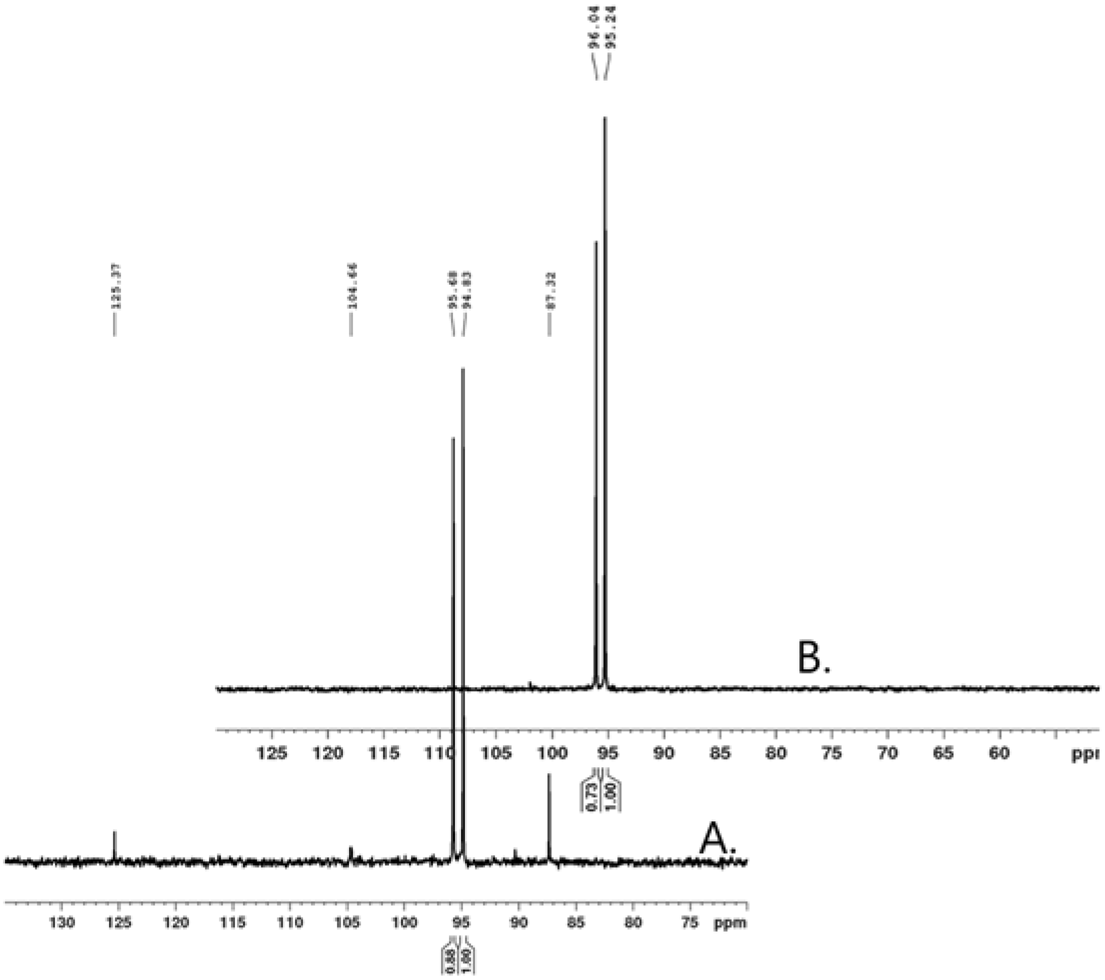

Figure S5. ^{31}P NMR spectra recorded after synthesis of 5’-*O*-DMT-*N*6-benzoyl-3’-amino-2’,3’-dideoxy-adenosine-3’-*N*-(2-thio-1,3,2-oxathiaphospholane).
Plot A: the crude reaction mixture; plot B: after „flash” chromatography.

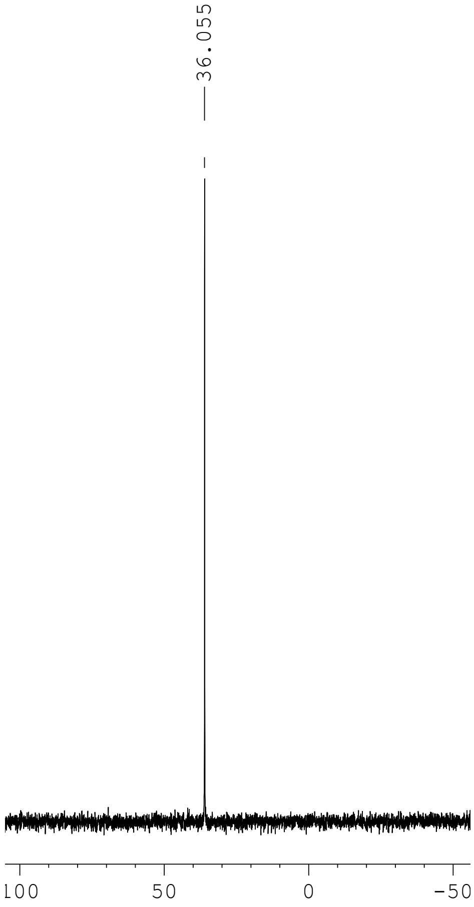

Figure S6.1. A ^{31}P NMR spectrum of $^{DMT}dG^{iBu}{}_{NPSMe}T_{OAc}$ amidodiester (**10f**).

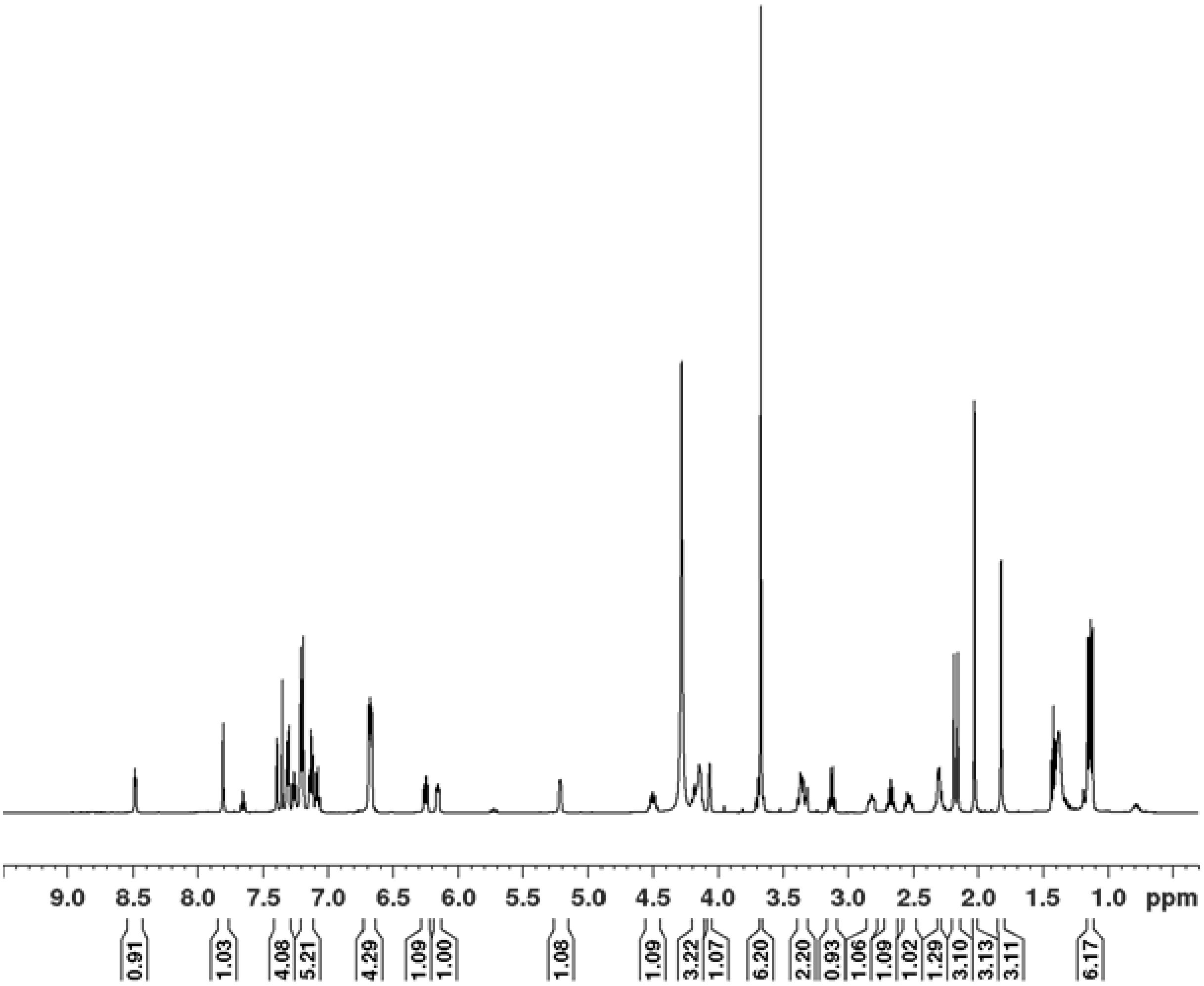

Figure S6.2. A ^{1}H NMR spectrum of $^{DMT}dG^{iBu}{}_{NPSMe}T_{OAc}$ amidodiester (**10f**).

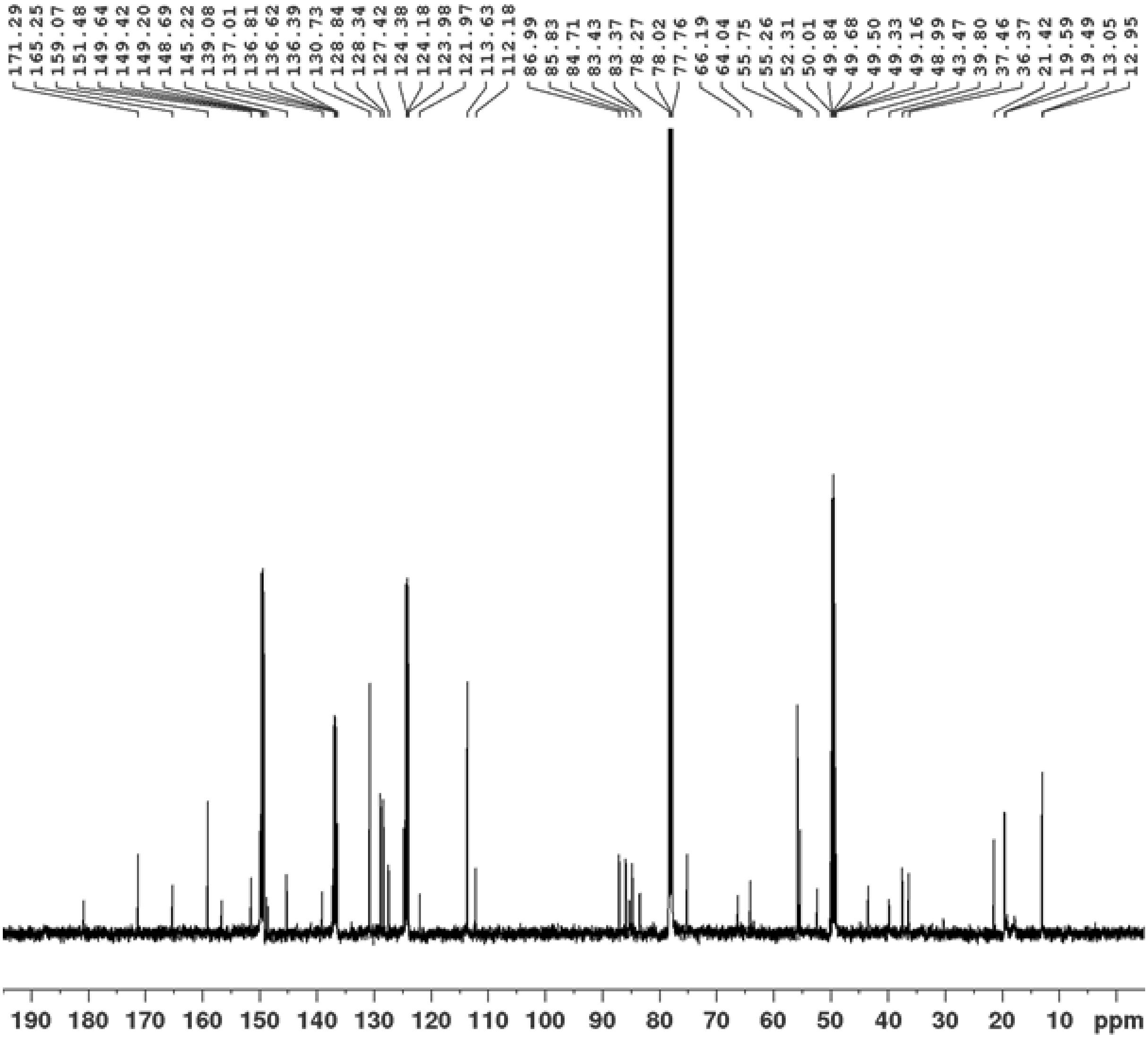

Figure S6.3. A ^{13}C NMR spectrum of $^{DMT}dG^{iBu}{}_{NPSMe}T_{OAc}$ amidodiester (**10f**).

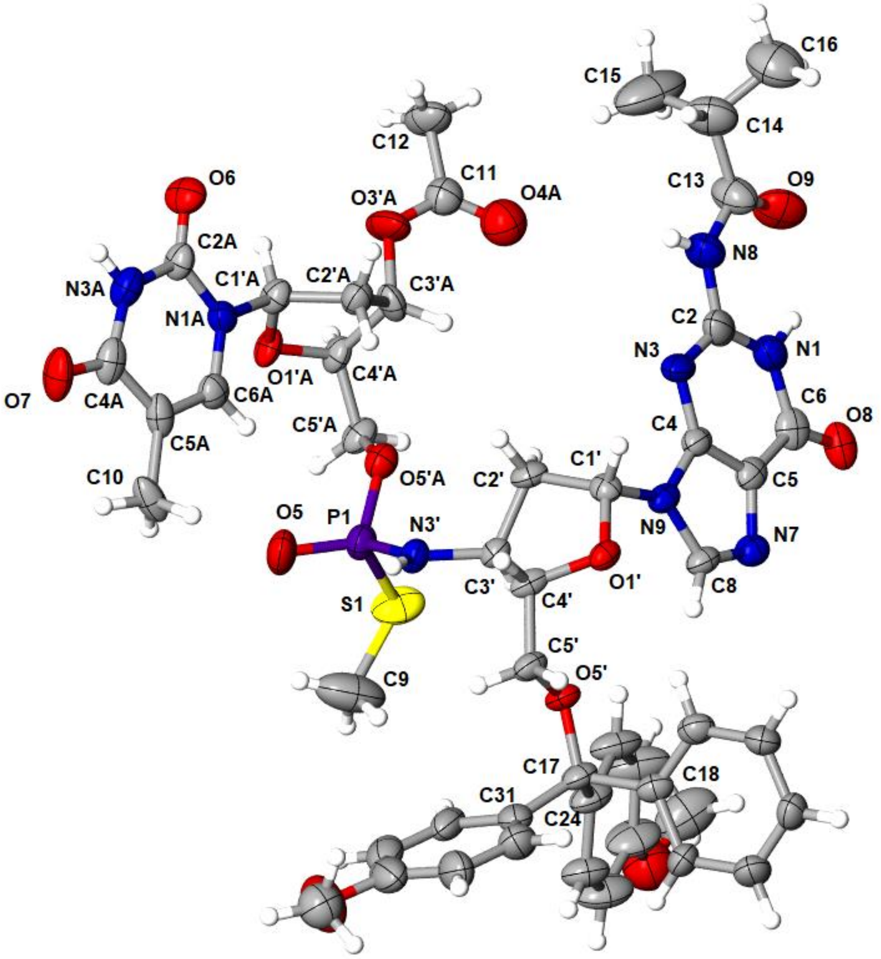

Figure S7. The structure of $^{DMT}dG^{iBu}{}_{NPSMe}T_{OAc}$ amidodiester (**10f**)
derived from the X-ray experiment. The atom labels ending with “A” indicate the atoms of the thymidine residue.

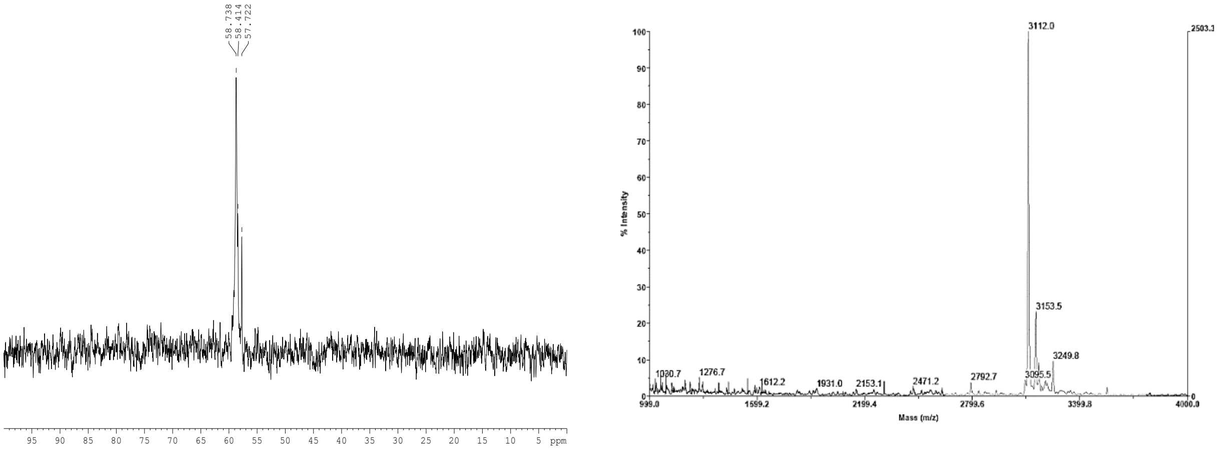

Figure S8. Analysis of **21** obtained from **5Tf**. Left: a ^{31}P NMR spectrum; right : a MALDI-TOF MS spectrum.

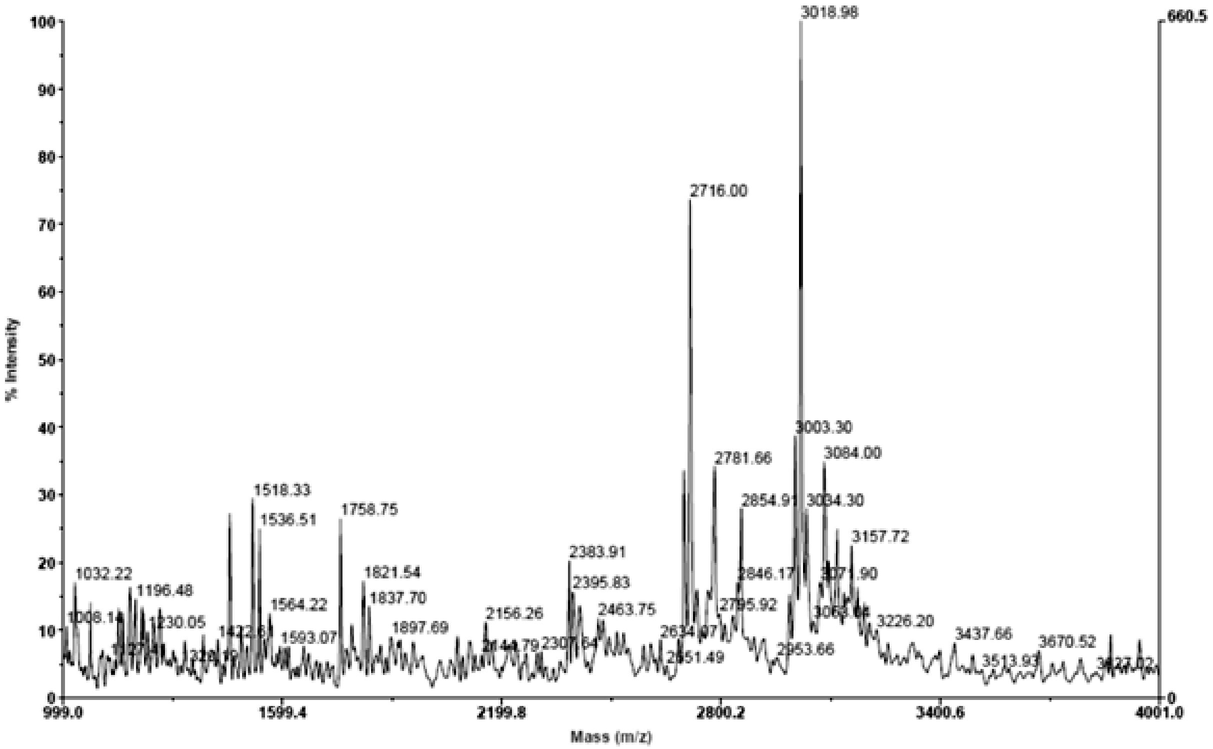

Figure S9. A MALDI-TOF MS spectrum recorded for $^{DMT}T_{PO}(T_{PO})_4(T_{NPS})_3T$.
The band at m/z 2716 corresponds to a molecular ion of the product detritylated due to the acidity of the matrix used.

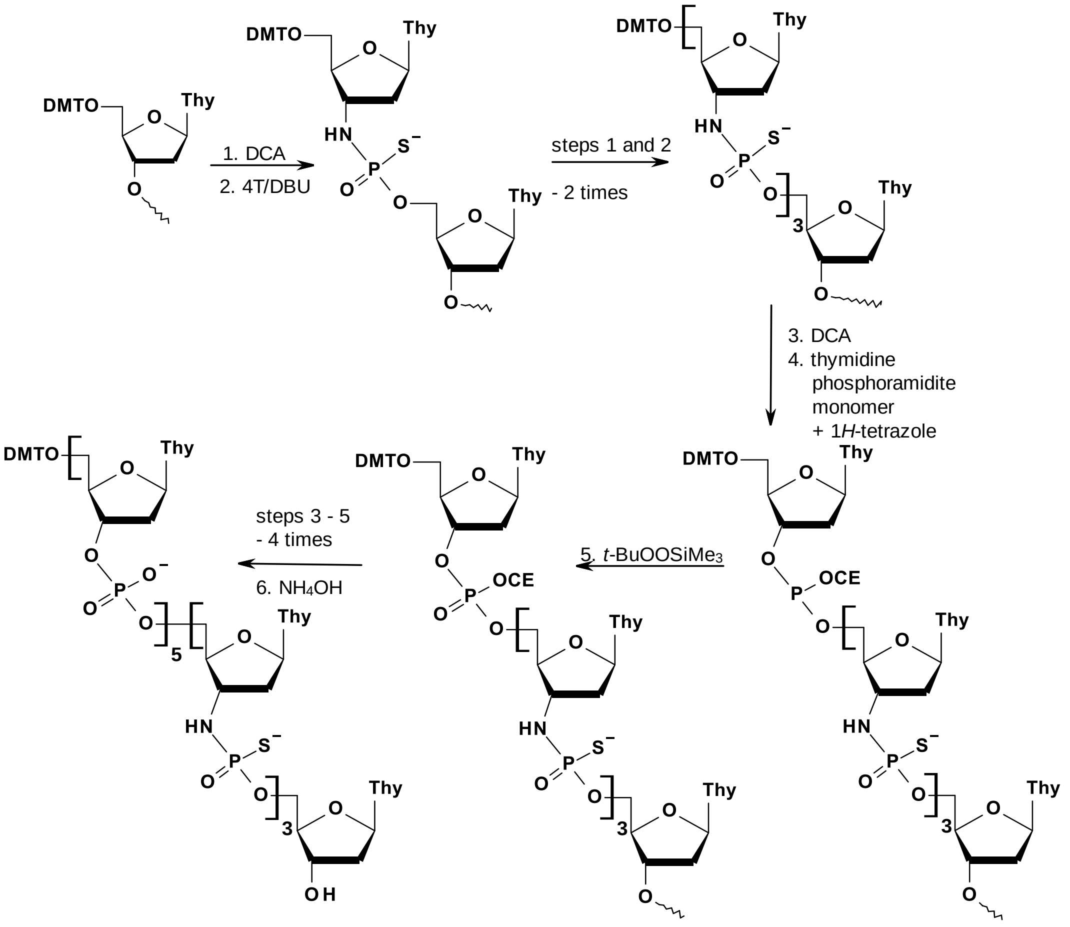

Scheme S1. Solid phase synthesis of chimeric NPS/PO oligomer $^{DMT}T_{PO}(T_{PO})_4(T_{NPS})_3T$ utilizing an unresolved $_N$OTP-T monomer **4T** and the standard thymidine phosphoramidite monomer.

Table S1. Isolated yield, HR MS or FAB MS (a negative ions mode), and ^{31}P NMR data for unresolved monomers **4-6**.

B′<br>R,R	Code	Yield<br>(isolated, %)	MW<br>Calc.<br>(Da)	FAB MS<br>(*m/z*)<br>$[M]^-$	^{31}P NMR<br><br>δ, ppm)
Cyt^{Bz}<br>H,H	**4C**	70	798	799.2385<br>HR MS,<br>$[M+H]^+$	96.45<br>95.94
Gua^{iBu}<br>H,H	**4G**	64	776	775	96.92<br>96.72
Thy<br>H,H	**4T**	71	681	680	95.31<br>94.66
Ade^{Bz}<br>Me,Me	**5A**	61	822	821	95.95<br>95.21
Gua^{iBu}<br>Me,Me	**5G**	49	804	803	97.04<br>96.64
Ade^{Bz}<br>$-(CH_2)_5-$	**6A**	58	862	861	97.01<br>96.56
Cyt^{Bz}<br>$-(CH_2)_5-$	**6C**	49	838	837	96.30<br>95.54
Thy<br>$-(CH_2)_5-$	**6T**	88	749	748	95.80<br>95.35

Table S2. Experimental details of crystallographic analysis.

**Crystal data**	
Chemical formula	$C_{48}H_{55}N_8O_{13}PS \cdot 2(CH_3OH)$
Mr	1079.11
Crystal system, space group	Monoclinic, P21
Temperature (K)	173
a, b, c (Å)	10.2758 (6), 14.8286 (8), 18.2612 (10)
β (°)	99.346 (3)
V (Å3)	2745.6 (3)
Z	2
Radiation type	Cu Kα
μ (mm−1)	1.41
Crystal size (mm)	0.24 × 0.05 × 0.03
**Data collection**	
Diffractometer	Bruker D8 Venture
Absorption correction	Multi-scan SADABS2016/2 (Bruker,2016/2)
No. of measured, independent and observed [I > 2σ(I)] reflections	57523, 10009, 9499
Rint	0.071
(sin θ/λ)max (Å−1)	0.603
**Refinement**	
R[F2 > 2σ(F2)], wR(F2), S	0.120, 0.370, 1.80
No. of reflections	10009
No. of parameters	773
No. of restraints	65
H-atom treatment	H atoms treated by a mixture of independent and constrained refinement
(Δ/σ)max	0.142
Δρmax, Δρmin (e Å−3)	1.18, −0.57
Absolute structure	Flack x determined using 4079 quotients [(I+)-(I-)]/[(I+)+(I-)] (Parsons, Flack and Wagner, Acta Cryst. B69 (2013) 249-259).
Absolute structure parameter	0.176 (7)
